# Identification of atrial myopathy and atrial fibrillation recurrence after ablation using 3D left atrial phasic strain from retrospective gated computed tomography

**DOI:** 10.1093/ehjimp/qyaf027

**Published:** 2025-03-06

**Authors:** Charles Sillett, Orod Razeghi, Tiffany M G Baptiste, Angela W C Lee, Jose Alonso Solis Lemus, Cristobal Rodero, Caroline H Roney, Ruibin Feng, Prasanth Ganesan, Hui Ju Chang, Paul Clopton, Nick Linton, Ronak Rajani, A J Rogers, Sanjiv M Narayan, Steven A Niederer

**Affiliations:** School of Biomedical Engineering and Imaging Sciences, King’s College London, London, UK; Cardiac Electro Mechanics Research Group, National Heart and Lung Institute, Imperial College London, London W12 0NN, UK; School of Biomedical Engineering and Imaging Sciences, King’s College London, London, UK; Department of Haematology, University of Cambridge, Cambridge, UK; School of Biomedical Engineering and Imaging Sciences, King’s College London, London, UK; Cardiac Electro Mechanics Research Group, National Heart and Lung Institute, Imperial College London, London W12 0NN, UK; School of Biomedical Engineering and Imaging Sciences, King’s College London, London, UK; Cardiac Electro Mechanics Research Group, National Heart and Lung Institute, Imperial College London, London W12 0NN, UK; School of Biomedical Engineering and Imaging Sciences, King’s College London, London, UK; Cardiac Electro Mechanics Research Group, National Heart and Lung Institute, Imperial College London, London W12 0NN, UK; Cardiac Electro Mechanics Research Group, National Heart and Lung Institute, Imperial College London, London W12 0NN, UK; School of Biomedical Engineering and Imaging Sciences, King’s College London, London, UK; School of Engineering and Materials Science, Queen Mary University of London, London, UK; Cardiovascular Institute, Stanford University, Stanford, CA, USA; Cardiovascular Institute, Stanford University, Stanford, CA, USA; Cardiovascular Institute, Stanford University, Stanford, CA, USA; Cardiovascular Institute, Stanford University, Stanford, CA, USA; Cardiac Electro Mechanics Research Group, National Heart and Lung Institute, Imperial College London, London W12 0NN, UK; School of Biomedical Engineering and Imaging Sciences, King’s College London, London, UK; Department of Cardiology, Guy’s and St Thomas’ NHS Foundation Trust, London, UK; Cardiovascular Institute, Stanford University, Stanford, CA, USA; Cardiovascular Institute, Stanford University, Stanford, CA, USA; School of Biomedical Engineering and Imaging Sciences, King’s College London, London, UK; Cardiac Electro Mechanics Research Group, National Heart and Lung Institute, Imperial College London, London W12 0NN, UK; Turing Research and Innovation Cluster in Digital Twins, Alan Turing Institute, London, UK

**Keywords:** atrial fibrillation, myocardial strain, retrospective gated computed tomography, catheter ablation

## Abstract

**Aims:**

Reduced left atrial (LA) mechanical function associates with atrial myopathy and adverse clinical endpoints in atrial fibrillation (AF) patients; however, conventional 2D imaging modalities are limited by atrial foreshortening and sub-optimally capture 3D LA motion.

**Objectives:**

We set out to test the hypothesis that 3D LA motion features from 4D (3D + time) retrospective gated computed tomography (RGCT) associate with AF phenotypes and predict AF recurrence in patients undergoing catheter ablation.

**Methods and results:**

Sixty-nine AF patients (60.8 ± 12.2 years, 39% female, 30% non-paroxysmal AF) who were indicated for CT coronary angiography including a RGCT protocol in sinus rhythm prior to ablation were included. We measured 3D LA endocardial motion by optimized 3D feature tracking and calculated 3D global and regional phasic strain and peak strain rates (SRs). AF recurrence was observed in 18 patients (26%) at 1-year. Global reservoir strain (*P* < 0.05) and contractile strain and SR (both *P* < 0.01) were reduced in patients with vs. those without recurrent AF. Global and anterior wall contractile SR were more predictive of recurrent AF than LA volume index (area under the curve, AUC: 0.74, 0.77, and 0.68, respectively). Reduced global conduit SR and septal reservoir strain were more strongly associated with non-paroxysmal AF than CHADS2-VASc (AUCs: 0.74, 0.75, and 0.59, respectively).

**Conclusion:**

Reduced passive and active 3D LA motion from 4D RGCT associates with more advanced AF and AF recurrence post-ablation, respectively. Future work should extend this approach to larger populations, with new low-radiation CT technologies to widen its applicability.

## Graphical Abstract

**Graphical Abstract:**
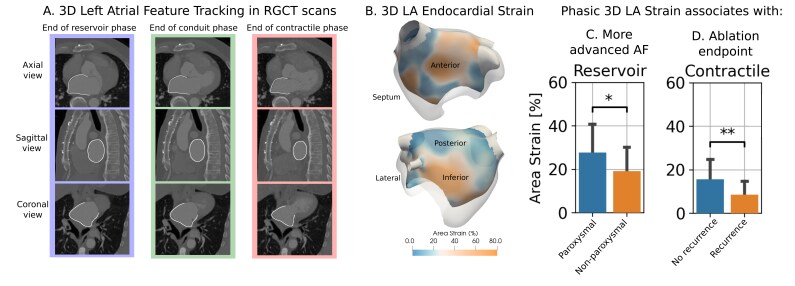
Features of 3D LA motion from retrospective gated computed tomography imaging identify adverse atrial phenotypes and AF recurrence after ablation. (*A*) 3D feature tracking of the LA endocardial contours overlaid on the CT frames over the cardiac cycle in the same axial, sagittal, and coronal views; (*B*) 3D area strain maps at the end of the reservoir phase, corresponding to maximum LA expansion, with overlaid range of 3D LA motion; (*C*) Lower reservoir strain in patients with non-paroxysmal AF compared with paroxysmal AF; (*D*) Lower contractile strain in patients with vs. without AF recurrence after ablation.

## Introduction

There is increasing focus on the need to identify patients with ‘early’ AF, in whom first-line anti-arrhythmic therapy may prolong survival and reduce morbidity.^1^ Atrial structural and electrical remodelling are associated with adverse clinical endpoints including more advanced AF, recurrence after catheter ablation and stroke.^2^ Atrial fibrosis, a hallmark of structural remodelling, is characterized by a proliferation of collagen and fibroblasts^3^ which leads to increased myocardial stiffness. Electrical remodelling leads to down-regulation of intracellular Ca^2+^ currents, which regulate contractile function^4^ and promotes AF. Atrial mechanical function may therefore reflect clinically relevant structural and electrical remodelling in AF patients, yet current methods are suboptimal for tracking atrial motion and biomechanics in 3D.

LA deformation is typically measured in the 2D apical two- and four-chamber imaging planes using speckle tracking echocardiography (STE) or cine magnetic resonance imaging (MRI). Despite success, 2D imaging approaches using STE and cine MRI suffer from atrial foreshortening, fail to address the complexities of 3D LA anatomy and motion^5,6^ and thus may potentially miss clinically relevant features of LA biomechanics. Issues with atrial foreshortening in 2D STE have been reported to overestimate atrial strain compared with 3D imaging.^7^ Measurement of 3D LA biomechanics is therefore motivated. Solutions using 3D echocardiography are limited by both challenges to image quality and technique availability,^8^ whereas standard cine MRI protocols have large slice thickness (4–8 mm) that makes accurate delineation of small-sized landmarks such as the pulmonary vein (PV) ostia (diameters as small as 9 mm),^9^ distances between adjacent PVs (<6 mm)^10^ and the proximity of the left superior PV to the LA appendage difficult in three dimensions. In contrast, standard 4D (3D + time) retrospective ECG-gated computed tomography (RGCT) scans produce 3D cine images of the cardiac anatomy with higher spatial resolution than both STE and MRI.^11^ RGCT images typically offer sub-millimetre in-plane resolution and slice thickness, which enables high-fidelity 3D images of the LA anatomy, circumvents image quality and resolution issues with STE and cine MRI, and therefore may be used to provide novel insights into 3D LA deformation.^6^

We hypothesized that measurement of 3D LA motion using 3D feature tracking in 4D RGCT scans may detect more advanced AF phenotypes and predict response to catheter ablation. Since both non-paroxysmal AF and recurrence after ablation have been associated with increased atrial fibrosis burden,^12,13^ we postulated that differences in 3D LA motion between AF type and ablation response may indicate changes in global and regional LA function associated with increased atrial remodelling. While global and regional features of 3D LA motion from 4D RGCT have recently been shown to associate with the presence of AF,^14^ it is unclear what features of 3D LA motion associate with the persistence of AF or predict AF recurrence post-ablation.

We set out to test whether novel features of 3D LA motion, measured from clinically indicated 4D RGCT scans, differ between (1) patients with non-paroxysmal vs. paroxysmal AF and (2) patients in whom AF recurred at 12 months after ablation compared with individuals without recurrence. We studied patients from the Stanford AF registry in whom we related global and regional strain and peak strain rates (SRs) to a rich repository of clinical variables to establish clinical phenotypes and clinical outcomes after ablation.

## Methods

### Study population

This was a single-centre retrospective study approved by our Institutional Review Board. We studied AF patients indicated for catheter ablation at Stanford University Hospital. Patients from the Stanford AF Registry who had been clinically indicated for coronary CT angiography that included a RGCT protocol prior to ablation were included. All patients provided written informed consent, and images were anonymized prior to analysis. Exclusion criteria were history of and prior procedures related to congenital heart disease, and absence of sinus rhythm during imaging. Patients not in sinus rhythm during the image acquisition were excluded, since we aimed to measure differences in atrial mechanical function due to inherent atrial remodeling and not rhythm during image acquisition. Paroxysmal AF was defined per guidelines^15^ as AF that self-terminated in <1 week. Patients with a history of persistent and long-standing persistent AF who were referred for ablation were grouped as having non-paroxysmal AF.^16^ No patients with permanent AF were included.

### Imaging protocol

RGCT scans were acquired using a CT scanner from either Siemens Healthcare (GmbH, Forchheim, Germany) or GE Healthcare (Chicago, IL, USA) using 99 ± 22 mL of intravenous contrast agents Isovue-300 or Isovue-370 (Bracco, Milan, Italy) with a flow rate of 5 mL/s. Scanner models are detailed in Supplementary data online, *Table S3*. Helical scanning was performed with a pitch between 0.15 and 0.33 and a gantry rotation time between 250 and 300 ms. Tube voltage (80/100/120/140 kV) and tube current between 43 and 318 mAs (reference values 200/234/244 mAs) were used. Scans had a median (interquartile range) CT dose index volume (CTDIvol) of 25.4 (16–42) mGy. Retrospective ECG gating at the time of original scan was used to reconstruct either 10 or 20 CT frames at 10 and 5% increments over the R-R interval, respectively. Rhythm during CT image acquisition was assessed from the ECG rhythm strip. In-plane isotropic spatial resolution ranged between 0.31 and 0.95 mm^2^ and slice thickness ranged between 0.625 and 2.0 mm.

### Image analysis

#### Volumetric assessment

LA and LV volumes were measured at each CT frame using an automatic segmentation tool.^17^ Automated segmentations were reviewed and manually corrected using an open-source application called CemrgApp^18^ (http://cemrgapp.com/). End-diastolic volumes (EDVs) and end-systolic volumes (ESVs) from the RGCT images were used to calculate CT-derived left atrial emptying fraction (LAEF), left ventricular ejection fraction (LVEF), and normalized to body surface area.

#### Patient-specific model creation

**Figure 1:**
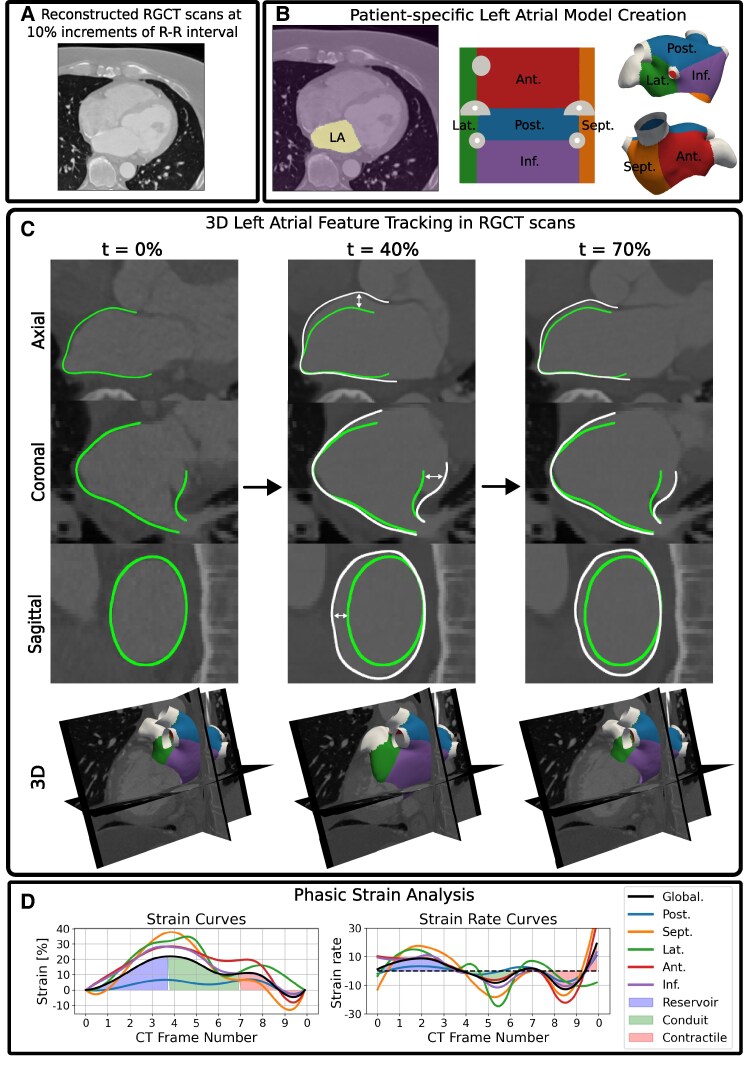
Pipeline for phasic 3D LA motion and strain measurement from RGCT scans. (*A*): Input data were ten reconstructed RGCT scans at 10% increments of the R-R interval. (*B*): Patient-specific LA models were created from the end- diastolic (*t* = 0%) CT frame. LA body regions were standardized using the UAC system. (*C*): 3D Feature tracking estimated 3D LA motion from the RGCT scans. The tracked 3D LA endocardial outline is shown at ventricular end-diastole (*t* = 0%), end-systole (*t* = 40%) and early diastole (*t* = 70%). The end-diastolic LA contour (in green) is repeated at later CT frames to explicitly outline the change in LA shape. White arrows outline LA enlargement at end-systole. (*D*): Global and regional phasic strain analysis from the 3D motion. Phases were defined using the global strain transient (black line). Ant, anterior; inf, inferior; lat, lateral; post, posterior; sept, septum.

Patient-specific LA endocardial surface models were semi-automatically created from the ED CT frame LA segmentation using CemrgApp^18,19^ (*Figure 1B* and Supplementary data online, *Figure S1*) which included labels for the PVs and left atrial appendage (LAA).^20^ Universal atrial coordinates (UACs) were calculated on each anatomy^21^ which enabled the separation of the LA body into five regions: septum, lateral, posterior, anterior, and inferior walls (see Supplementary data online, *Figure S2*) for regional strain measurement.

#### Feature tracking

We have previously developed a workflow to analyse 3D LA motion from RGCT scans.^14^ Three-dimensional LA feature tracking, optimized for the application of LA motion estimation in RGCT images,^14^ enabled 3D LA motion estimation. The number of reconstructed frames may impact strain measurement,^22^ therefore, ten RGCT images at 10% increments were used for LA feature tracking across all cases, since not all cases had 20 images reconstructed at 5% increments. The effect of 10 vs. 20-frame feature tracking on global strains was compared in a subset test cohort.

### Strain measurement

Three-dimensional LA motion was quantified using area strain, which computes the percentage area change of each local surface element with respect to the end-diastolic anatomy. Positive and negative area strain denote endocardial expansion and contraction, respectively.

#### Regional strain measurement

The LA body, excluding the PVs and LAA, was divided into five regions using the UACs (*Figure 1B*). Global and regional area strain measurements were calculated using the mean elemental area strains over the entire LA body surface and within each region, respectively.

#### Phase definition

Atrial functional phases—reservoir, conduit, and contractile—were automatically identified from the global area strain curve, and phasic strains and peak SRs were extracted (see Supplementary data online, *Figure S4*). As recommended in the consensus document from the EACVI/ASD/Industry Task Force on echocardiographic deformation imaging,^23^ minimum LA strain was defined as end-diastole. The conduit-contractile phase boundary was defined by identifying an inflection point where the strain curve levels off after the sharp conduit downward slope after peak reservoir strain. The first-time derivative of the strain curves with respect to normalized time enabled SR calculation. All phase separations were reviewed manually, verified with the LA volume curve, and corrected if necessary to ensure consistency with the recommendations in Badano et al.^23^ Regional phasic measurements were extracted from the regional strain and SR curves using the global phase definitions.

### Ablation and detection of recurrence on follow-up

Patients underwent either a pulmonary vein isolation (PVI) only, ablation of extra-PV sites if PVs were previously isolated, or PVI and ablation of extra-PV sites including potential driver sites. Patients were followed up at 12 months post-ablation by clinic visits, phone call, or device check. In follow-up, rhythm was determined from patient history and symptoms and cumulative arrhythmia monitoring including 12-lead ECGs, ambulatory ECG monitors or implantable loop recorders. Recurrence was defined as per AF ablation guidelines at the time of data collection^24^ as >30 s of atrial arrhythmias detected on cumulative arrhythmia monitoring.

### Statistical analysis

Continuous variables are presented as mean ± SD. Categorical variables are presented as frequencies and percentages. Differences between group means were evaluated using two-sided Student’s *t*-test (continuous variables) and either χ^2^ or Fisher’s exact test (categorical variables) as appropriate. Mixed ANOVA was used to test for the interaction between regional parameters with AF recurrence and AF type. For global parameters that differed significantly between groups, differences in regional parameters were qualitatively explored. Receiver operator characteristic (ROC) analysis was used to investigate the association of global and regional parameters with AF type and recurrence. The bootstrap method was used to construct 95% confidence intervals in area under curve (AUC) values. DeLong’s test was used to assess differences between ROC curves. K-means clustering was used to identify patient clusters with similar 3D LA motion. Statistical analysis was completed in Python using the scipy, scikit-learn, and pingouin libraries. We used an alpha level of 0.05.

## Results

Out of a total of 75 patients initially identified, 6 were excluded due to incomplete imaging, leaving 69 AF patients who were included in the study cohort. Baseline differences between patients with paroxysmal vs. non-paroxysmal AF are displayed in Supplementary data online, *Table S8*. Differences between patients with first-time vs. repeat AF ablation are displayed in Supplementary data online, *Table S13*. Occurrence of recurrent AF was observed in 18 (26.1%) patients within 12-month follow-up. The clinical characteristics of patients separated by the occurrence of recurrent AF are shown in *Table 1*.

**Table 1:** Clinical characteristics of the study population separated by occurrence of recurrent AF.

Variable	AF recurrence (*n* = 18)	No AF recurrence (*n* = 51)	*P*-value
Age, years	62.5 (14.4)	60.2 (11.4)	0.486
Female, n (%)	10 (55.6)	17 (33.3)	0.168
Prior AF Ablation, n (%)	10 (55.6)	22 (43.1)	0.526
Height (cm)	174.2 (13.5)	175.5 (11.9)	0.706
Weight (kg)	94.3 (19.3)	89.2 (21.1)	0.369
BMI	31.1 (5.2)	28.8 (5.4)	0.120
CHADS2-VASc	2.6 (1.5)	1.9 (1.6)	0.124
CHADS2-VASc ≥ 2, n (%)	14 (77.8)	28 (54.9)	0.153
Arrhythmia history			
Duration of AF (days)	1823 (880–2605)	1855 (652–3342)	0.960
Paroxysmal AF, n (%)	13 (72.2)	35 (68.6)	1.0
Non-paroxysmal AF, n (%)	5 (27.8)	16 (31.4)	1.0
Comorbidities			
Coronary artery disease, n (%)	2 (11.1)	8 (15.7)	1.0
Congestive heart failure, n (%)	3 (16.7)	7 (13.7)	0.713
Diabetes mellitus, n (%)	3 (16.7)	8 (15.7)	1.0
Hypertension, n (%)	13 (72.2)	27 (52.9)	0.251
Hyperlipidemia, n (%)	7 (38.9)	22 (43.1)	0.971
Valvular disease, n (%)	0 (0.0)	4 (7.8)	0.566
Ablation strategy			
PVI ablation only, n (%)	9 (50.0)	30 (58.8)	0.709
Extra-PV ablation only, n (%)	3 (16.7)	5 (9.8)	0.421
PVI and extra-PV ablation, n (%)	6 (33.3)	16 (31.4)	1.0
Arrhythmia medications			
Amiodarone	9 (50.0)	30 (58.8)	0.709
Beta-blocker	3 (16.7)	5 (9.8)	0.421
CCBs	6 (33.3)	16 (31.4)	1.0
Digoxin	9 (50.0)	30 (58.8)	0.709
Dofetilide	3 (16.7)	5 (9.8)	0.421
Dronedarone	6 (33.3)	16 (31.4)	1.0
Flecainide	9 (50.0)	30 (58.8)	0.709
Propafenone	3 (16.7)	5 (9.8)	0.421
Quinidine	6 (33.3)	16 (31.4)	1.0
Sotalol	9 (50.0)	30 (58.8)	0.709

CCB, calcium channel blocker.

### CT image analysis

*Table 2* presents a comparison of CT-derived volumetric parameters for the LA and LV between endpoints. Supplementary data online, *Table S9* presents this information between AF types. CT-derived LA volumes and LAEFs were significantly larger and smaller, respectively, in the non-paroxysmal AF group vs. the paroxysmal AF group (*P* < 0.05) and in the recurrent AF vs. the recurrence-free group (*P* < 0.05). There were no differences in LV parameters when separating by AF type or endpoint.

**Table 2:** Comparison of CT volumetric parameters between recurrence and no recurrence of AF

Variable	AF recurrence (*n* = 18)	No AF recurrence (*n* = 51)	*P*-value
CT LA parameters			
LA EDV [mL]	110.2 (48.2)	82.3 (35.7)	**0.011**
LA ESV [mL]	147.1 (48.3)	126.5 (36.5)	0.063
LA EDVI [mL/m^2^]	52.9 (22.8)	41.0 (19.8)	**0.040**
LA ESVI [mL/m^2^]	70.7 (22.8)	62.5 (19.1)	0.138
LA SV [mL]	41.4 (18.0)	49.0 (19.0)	0.147
LAEF [%]	29.9 (14.4)	38.4 (13.8)	**0.029**
CT LV Parameters			
LV EDV [mL]	183.6 (53.0)	183.7 (33.3)	0.992
LV ESV [mL]	92.9 (33.6)	92.0 (23.1)	0.895
LV EDVI [mL/m^2^]	87.0 (16.6)	90.1 (12.8)	0.418
LV ESVI [mL/m^2^]	43.8 (10.9)	45.1 (10.5)	0.664
LV SV [mL]	90.7 (24.0)	91.7 (19.7)	0.854
LVEF [%]	49.7 (6.5)	50.1 (6.9)	0.818

Bold *P*-values indicate statistically significant results.

EDV, end-diastolic volume; EDVI, end-diastolic volume index; ESV, end-systolic volume; ESVI, end-systolic volume index; LA, left atrium; LAEF, left atrial emptying fraction; LV, left ventricle; LVEF, left ventricular ejection fraction; SV, stroke volume.

### Differences in CT-derived phasic strain

**Figure 2:**
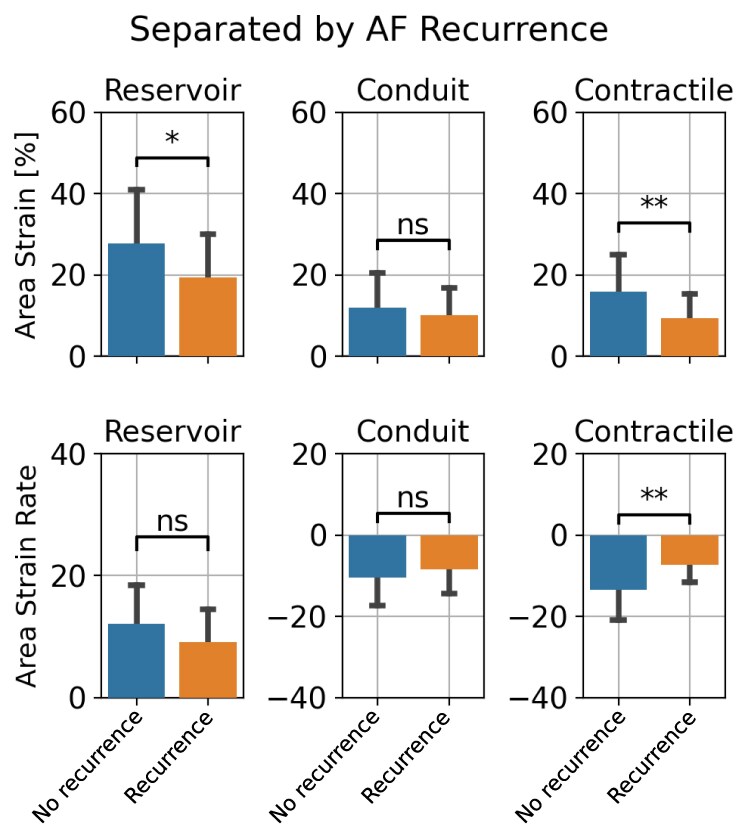
Comparison of the global phasic strains and peak SRs from 3D LA motion between AF recurrence vs. no recurrence. Contractile parameters displayed the most significant differences in patients with successful AF ablation endpoint. *P*-value legend, ns: not significant; *: *P* < 0.05, **: *P* < 0.01.

*Figure 2* displays the comparisons of global phasic strains and peak SRs between patients separated by AF recurrence. *Table 3* details differences in global phasic strains and peak SRs between patients separated by AF type. Supplementary tests found that 10-frame feature tracking led to similar phasic strains (mean absolute difference < 1.2%) and lower phasic SRs (mean absolute difference < 7% per normalized time) compared with 20-frame feature tracking, which is likely due to the differences in sampling rate. Therefore, feature tracking for all cases was performed using 10 reconstructed frames to avoid strain differences due to differences in sampling rate.

**Table 3:** Comparison of the global phasic strains and peak SRs from 3D LA motion between paroxysmal and non-paroxysmal AF groups

Variable	Non-paroxysmal AF (*n* = 21)	Paroxysmal AF (*n* = 48)	*P*-value
CT LA strains (%)			
Reservoir	28.2 (13.0)	19.6 (11.2)	**0.010**
Conduit	12.7 (8.9)	8.7 (5.2)	0.064
Contractile	15.6 (8.5)	10.9 (9.0)	**0.041**
CT LA strain rates (% per normalized time)			
Reservoir	12.6 (6.0)	8.5 (5.6)	**0.010**
Conduit	11.2 (7.1)	7.0 (4.9)	**0.014**
Contractile	13.1 (7.3)	8.9 (6.7)	**0.027**

Bold *P*-values indicate statistically significant results.

#### AF type

The global reservoir strain and reservoir and conduit SRs exhibited the most significantly reduced magnitudes in the non-paroxysmal vs. paroxysmal AF group (*Table 3*). Global contractile strain and SR exhibited smaller, but significant reductions in means in the non-paroxysmal AF group.

#### AF recurrence

The global contractile parameters were the most significantly reduced parameters in the AF recurrence group compared with the recurrence-free group (contractile strain: 9.3 ± 6.0% vs. 15.9 ± 9.1%, *P* = 0.006; contractile SR: −6.8 ± 4.4 vs. −13.1 ± 7.7, *P* = 0.002), *Figure 2*. Reservoir strain exhibited a smaller, but significant, reduction in the recurrent AF group (*P* < 0.05).

### Discriminative ability of CT-derived phasic strains to predict clinical phenotypes

**Figure 3:**
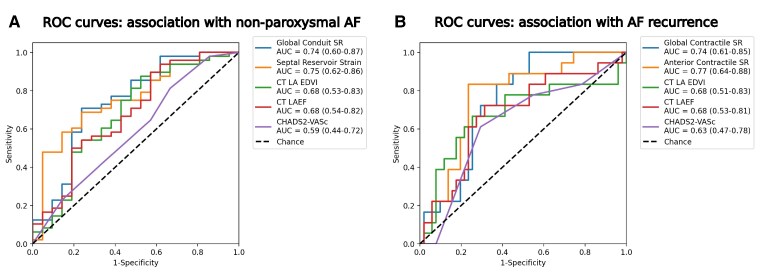
ROC analysis compares predictive ability of conventional and RGCT-derived 3D LA biomechanics features to identify (*A*) non-paroxysmal AF and (*B*) AF recurrence. Mean and 95% CIs for AUC values are presented for the most predictive global and regional features from RGCT-derived 3D LA biomechanics and conventional features. Passive and contractile features separated non-paroxysmal AF and recurrent AF, respectively.

*Figure 3* displays the ROC analysis for the association of features with (*A*) non-paroxysmal AF and (*B*) AF recurrence. The ROC curves for the most predictive global and regional 3D LA motion features with the highest AUCs are shown in *Figure 3*. The full list of AUC values for all features is listed in Supplementary data online, *Tables S10* and *S11*.

#### AF type

**Figure 4:**
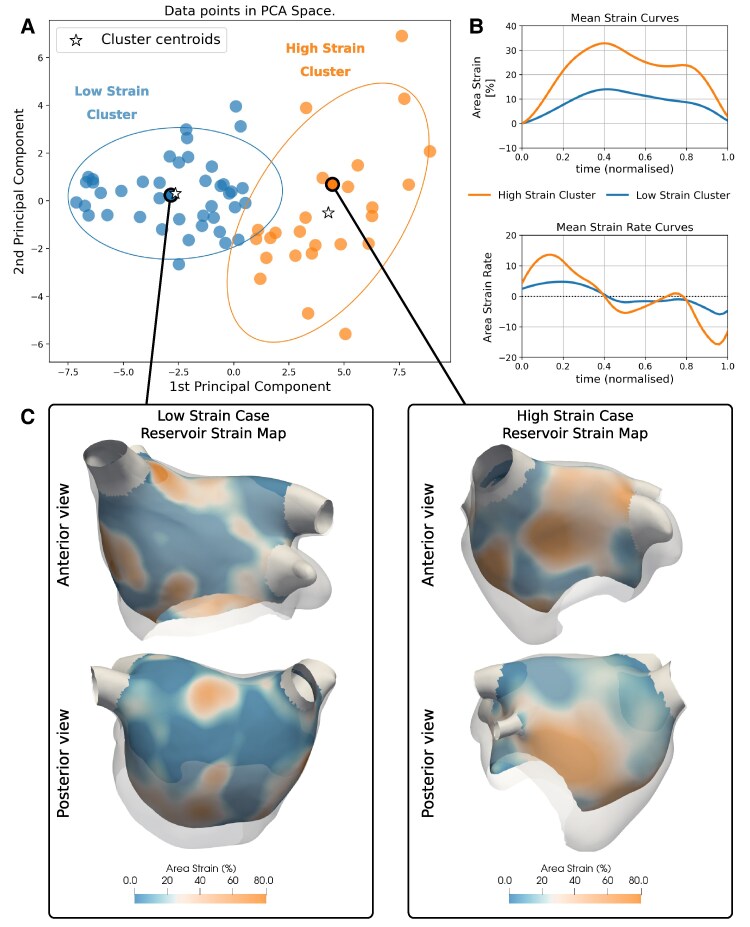
Results of K-means clustering using the RGCT-derived 3D strain and SR parameters. (*A*): The two identified clusters shown in the first two principal components’ space with 95% CI ellipses. No clinical variables were used to identify the clusters. (*B*): Mean strain (top) and SR (bottom) curves in both clusters. (*C*): Reservoir strain maps from representative cases within the clusters. Extent of 3D LA motion at ventricular end-systole is shown by the semi-transparent shell. Blue and orange represent smaller and larger LA stretch and hence reservoir strain, respectively.

The global conduit peak SR gave a higher AUC than LA EDVI, LAEF, and CHA2DS2-VASc for predicting non-paroxysmal AF (AUCs: conduit SR: 0.74; LA EDVI: 0.68; LAEF: 0.68; CHA2DS2-VASc: 0.59) (*Figure 4A*). The most predictive regional parameters were related to the reservoir and conduit phases (see Supplementary data online, *Table S10*). Out of the regional parameters, the septal wall reservoir strain and SR were the most predictive regional parameters for non-paroxysmal AF (AUC = 0.75 for both).

#### AF recurrence

The global contractile SR was superior for predicting AF recurrence post-ablation than LA EDVI, LAEF, and CHA2DS2-VASc (AUCs: contractile SR: 0.74; LA EDVI: 0.68; LAEF: 0.68; CHA2DS2-VASc, 0.63) (*Figure 4B*). The most predictive regional parameters were also related to the contractile phase (see Supplementary data online, *Table S11*). Out of the regional parameters, the anterior wall contractile SR was the most predictive regional parameter with an AUC of 0.77, a positive predictive value of 0.56, sensitivity of 0.83, specificity of 0.76, and F-1 score of 0.67.

### Unsupervised machine learning

K-means clustering was used to identify groups of patients with similar 3D LA motion. An outlier identified as the sole case in a cluster was removed (see Supplementary data online, *Figure S10*) and clustering was repeated. Silhouette score analysis showed that the optimal number of clusters was two (see Supplementary data online, *Figure S11*). *Figure 4* displays results of K-means clustering using all 36 CT-derived strain and SR parameters (three phases; strain and SR; global and five regional parameters) in the space defined by the first two principal components (*Figure 4A*), which captured 58% of the data variance. Mean strain and SR curves within each cluster demonstrate the identified clusters were characterized by low and high LA biomechanics (*Figure 4B*). Reservoir strain maps and a range of 3D LA motion (overlaid as a semi-transparent 3D shell) of representative cases near to the cluster centroids are shown in *Figure 4C*.

There were no significant differences in BMI, comorbidities and LVEF between clusters (see Supplementary data online, *Table S12*). The low-strain cluster had significantly higher age, more prevalent female sex and prior ablation, and a higher AF recurrence rate (all *P* < 0.05) than the high-strain cluster. CHADS2-VASc score and prevalence of non-paroxysmal AF were both higher in the low-strain cluster but lacked statistical significance. The low-strain cluster was characterized by larger LA EDVs and lower LAEFs (*P* < 0.001).

### Secondary analysis: prior ablation

Secondary analysis was performed to investigate if features of 3D LA motion identified patients with prior ablation. Global contractile strain and SRs and reservoir strain were significantly reduced in the repeat ablation group (all *P* < 0.05) (see Supplementary data online, *Figure S12*) and the posterior wall, which includes the area around the PVs, exhibited the most marked reduction in contractile phase parameters (see Supplementary data online, *Figure S13*). ROC analysis showed the posterior wall contractile strain and reservoir SR gave significantly improved prediction of prior ablation than the corresponding global parameters (*P* < 0.05 using DeLong’s test) (see Supplementary data online, *Tables S16* and *S17*).

## Discussion

This study reveals that novel features of 3D LA motion, measured from clinically indicated 4D RGCT scans, associate with AF persistence and recurrent AF post-ablation. We found, first, that reduced global reservoir and conduit SR, governed by passive atrial biomechanics, were the most strongly associated parameters with non-paroxysmal vs. paroxysmal AF. Secondly, that reduced global contractile strain and SR, governed by active atrial biomechanics, were most strongly associated with recurrent AF post-ablation. Atrial 3D biomechanics also tracked several features of ‘atrial substrate’ such that patients with low-strain were older, more likely to be female, had a higher prevalence of prior ablation, and had a higher rate of AF recurrence than those with preserved strain parameters. To our knowledge, this work represents the largest study of 3D LA motion measured from clinically indicated RGCT imaging in AF patients.

### Association of phasic 3D LA mechanics with AF persistence and AF recurrence

All patients were in sinus rhythm during imaging, and there were no differences in LVEF between groups, which suggests that differences in 3D LA biomechanics were due to local atrial dysfunction and remodelling and not due to transient AF episodes or LV systolic dysfunction.

#### AF persistence

Patients with non-paroxysmal AF often have <100% AF burden,^25^ which enabled us to identify patients with a history of persistent or long-standing persistent AF in sinus rhythm during image acquisition. Our findings show that more advanced AF may be associated with 3D indices of passive LA biomechanics, reflecting reduced atrial compliance and suggesting greater atrial myocardial stiffness consistent with fibrosis. Interstitial atrial fibrosis develops at 6–12 months after AF onset,^16^ and persistent AF has been characterized with greater fibrosis burden by late gadolinium enhancement.^12^ These findings, to our knowledge, are the first to report differences in 3D LA biomechanics associated with more advanced AF and are consistent with previous reports of lower global longitudinal strain in persistent vs. paroxysmal AF^12^. Furthermore, we found that septal wall passive biomechanics parameters were the regional parameters most strongly associated with more advanced AF, which may be explained by increased stiffness and reduced compliance due to the proximity of the adjacent right atrium.

#### AF recurrence

In contrast to AF persistence, post-ablation recurrent AF was most strongly separated by reduced atrial contractile function. Reduced LA contraction may reflect the degree of electrical remodelling, due to down-regulation of Ca^2+^ ion channels that regulate myocardial contraction.^26^ The DECAAF study associated fibrosis burden with AF recurrence post-ablation.^13^ Fibrosis can present as both interstitial fibrosis, which predominantly impairs passive function, and replacement fibrosis, which impairs active contraction.^27^ Our findings may indicate that increased atrial replacement fibrosis and the corresponding decreased contractility predict recurrent AF post-ablation.

Whilst baseline LA mechanical dysfunction in patients with recurrent AF post-ablation has previously been reported with STE^28^ and cine MRI,^29^ these studies measured LA biomechanics in 2D, which is limited by atrial foreshortening and in its regional assessment of atrial dysfunction. In this study, 3D motion analysis enabled by 4D RGCT scans with 3D feature tracking showed that the anterior wall contractile peak SR was the most predictive regional parameter for recurrent AF. This could be due to the anterior wall's proximity to the LAA and the mitral annulus, which both exhibit large regional myocardial deformation,^6^ leading to high negative peak SR^30^ and larger differences between patient subgroups separated by endpoint.

The degree of remodeling in the body of the atria may reflect an additional arrhythmogenic substrate not targeted by PVI. Global and regional contractile parameters gave greater AUC values for predicting AF recurrence than LA volume, which is consistent with previous reports of the incremental value of LA strain for AF recurrence prediction over LA enlargement.^29^ Our finding that atrial contractile function was the most predictive parameter of AF recurrence post-ablation is consistent with previous studies that used echocardiography and cine MRI.^29,31^ We additionally found similar predictive ability of RGCT-derived strain (AUC, 0.74) compared with cine MRI^29^ (AUC, 0.70), 2D STE^32^ (AUC, 0.70), and 3D STE^33^ (AUC, 0.73).

### LA strain and prior ablation

We found reduced global passive and active LA biomechanics in patients who had previously underwent AF ablation compared with those imaged prior to first-time ablation. Furthermore, the posterior wall reservoir SR and contractile strain parameters gave significantly improved prediction of prior ablation than corresponding global parameters which implies a preferential regional decrease in mechanical viability in the most common target area for ablation, as expected. Our findings were consistent with studies that found ablation alters the mechanical behaviour of the LA and negatively impacts atrial diastolic and systolic function.^34^ Future investigation into atrial strain differences that associate with time since prior ablation or procedural differences is motivated to investigate further how AF ablation impacts post-procedural atrial function.

### Clustering using 3D mechanical biomarkers

Clustering analysis identified groups of patients with low and high 3D LA motion and demonstrated that lower LA mechanical function alone identified a low-strain cluster with significant LA enlargement and greater rate of AF recurrence. Previous studies have employed clustering techniques to identify patients with differing cardiovascular risk^35^ and ventricular diastolic dysfunction^36^ using LA strain. These, however, were based upon 2D strain measurements from STE, in contrast to our study, where 3D LA motion features were used. Ntalianis *et al*.^35^ clustered based on time-series analysis using the entire shape of the LA strain curves, whereas in our study, more interpretable phasic features from global and regional measurements were used. Carluccio *et al*.^36^ used LA strain in addition to diastolic-related clinical variables to identify clusters. In this study, only features of LA mechanical function were used as clustering input to identify risk factors.

### Clinical utility of 4D computed tomography-derived LA motion

Our study provides proof-of-principle results that global and regional features of 3D LA motion from 4D RGCT scans are associated with more advanced AF and recurrent AF post-ablation and motivates studies to define novel phenotypes.

The high spatial resolution of RGCT makes it better suited than echocardiography and cine MRI to image 3D LA biomechanics due to the complexity of the 3D LA anatomy. Atrial foreshortening and poor signal-to-noise ratio limit 2D echocardiographic techniques, whereas typical diameters of PV ostia (as small as 9 mm),^9^ distances between adjacent PVs (<6 mm)^10^ and the proximity of left superior PV to the LAA make accurate delineation of the LA in 3D using cine MRI (4–8 mm slice thickness) difficult. In contrast, RGCT produces multiple high spatial resolution 3D CT images over the cardiac cycle, with slice thickness on the same or lower order of magnitude than typical LA wall thickness, which makes it ideal to image the LA anatomy.^11^ Moreover, RGCT benefits from more rapid image acquisition times than cine MRI,^5^ is less dependent on skilled operators for probe placement in echocardiography,^6^ and uses a modality that is often used as part of pre-procedural imaging for AF ablation. To our knowledge, this work is the first description of using RGCT to measure 3D LA mechanical function in the reservoir, conduit, and contractile phases, and that also relates these features to AF type and recurrence post-ablation.

Whilst we acknowledge that wide clinical use of RGCT protocols is limited by the associated radiation dose (median dose in this cohort: 25.4 mGy), new low-dose protocols could address this issue and widen the clinical applicability of RGCT.^37^ Manohar *et al*.^37^ report an additional effective dose of only 2.8 mSv to include a whole-cycle RGCT protocol with a coronary CT angiography acquisition.^36^ Furthermore, a recent radiation dose survey found a 78% reduction in dose with CT angiography^38^ between 2007 and 2017, which is likely to reduce further in the future with more dose-efficient scan protocols and, potentially, the advent of photon counting CT.^39^ Our retrospective study, which included AF patients indicated for RGCT as part of coronary CT angiography for treatment planning prior to AF ablation, therefore provides proof-of-principle results that demonstrate 3D LA strain can be measured from standard RGCT scans, which may prove useful in assessing future risk of AF recurrence post-ablation. Given potential future lower-dose CT, the methods used in this study could be applied to a wider indication of CT imaging.

### Study limitations

Firstly, while this is one of the larger reported cohorts of 4D RGCT for 3D LA motion assessment, this remains a single-centre, retrospective study, and future studies should further enlarge the cohort of AF patients at multiple centres to improve generalizability. Such studies are planned. Secondly, while RGCT is not routinely indicated for AF ablation patients to minimize radiation burden, our study utilized RGCT that was acquired for clinical indications such as valvular sizing for transcatheter aortic valve replacement. Although aortic stenosis may introduce diastolic dysfunction, which could affect atrial biomechanics, we found no substantial difference in LVEF when separating by AF type or endpoint (*Table 2* and Supplementary data online, *Table S9*). Third, although the presented analysis of RGCT images enabled a novel regional analysis of 3D atrial biomechanics, we found no significantly superior prediction of non-paroxysmal AF or AF recurrence using regional parameters vs. corresponding global parameters with a DeLong’s test. This should be noted when discussing regional parameters as superior predictors of AF type or endpoint.

Fourth, RGCT typically has fewer reconstructed temporal frames than other modalities used for LA strain measurement. However, we found that 10 frames reconstructed at 10% increments of the R-R interval were sufficient to qualitatively visualize active LA contraction, which was apparent in the 3D LA strain and volume curves. Twenty frame RGCT reconstructed at 5% intervals using new low-dose CT technology could improve the ability of RGCT to temporally visualize the conduit-contractile phase boundary. Finally, while radiation dosage of current imaging sequences may limit the applicability of this RGCT approach, new FDA-approved low-radiation dose CT^37^ and, potentially, photon counting CT technology could reduce the radiation burden of RGCT and widen its indication.

### Conclusions

We have shown that active and passive 3D LA motion derived from clinically indicated RGCT scans is associated with more advanced AF, recurrent AF post-ablation and prior AF ablation. This method has the potential to non-invasively probe atrial fibrosis burden using local 3D mechanical dysfunction. Future low-dose CT technology could widen the clinical applicability of RGCT.

## Supplementary Material

qyaf027_Supplementary_Data

## Data Availability

The datasets presented in this article are not readily available to be shared publicly due to the sensitive nature of patient data. Reasonable requests to access the datasets should be directed to the corresponding author, C.S.

## Lead author biography

Charles Sillett is a PhD candidate in the Biomedical Engineering and Imaging Science department at King’s College London. He is also a visiting student at the National Heart and Lung Institute at Imperial College London. His current field of interest is three-dimensional whole-heart cardiac biomechanics measurement and how this relates to AF, aortic stenosis and heart failure.
