# Supplementary material for: Identification of atrial myopathy and atrial fibrillation recurrence after ablation using 3D left atrial phasic strain from retrospective gated computed tomography
Source: Eur Heart J Imaging Methods Pract. 2025 Mar 6;3(1):qyaf027. doi: 10.1093/ehjimp/qyaf027 (PMC11959267; doi:10.1093/ehjimp/qyaf027)
Supplement: qyaf027_Supplementary_Data [file qyaf027_supplementary_data.docx]

**Supplemental Material Contents**

**Methods**

1. **Patient-Specific Model Creation**
   1. **Segmentation and Mesh Creation**
   2. **LA Region Definition**
2. **Feature Tracking**
3. **Strain Measurement**
   1. **Phase Definition**
4. **CT Scanner Details**

**Results**

1. **Mixed ANOVA Results**
   1. **AF Type**
   2. **AF Recurrence**
2. **Differences in Strain and Image Analysis**
   1. **AF Type**
   2. **AF Recurrence**
3. **Receiver Operating Characteristic Analysis**
   1. **AF Type**
   2. **AF Recurrence**
4. **Unsupervised Machine Learning Clustering**
5. **Secondary Analysis: Prior Ablation**
   1. **Mixed ANOVA Results**
   2. **Global and Regional Strain Differences**
   3. **ROC Analysis**
      1. **DeLong Analysis for Prior Ablation Classification**
6. **Sample Size Power Analysis**
7. **Group Overlap Analysis**

**Methods**

**1 Patient-specific Model Creation**

Patient-specific models of the left atrial (LA) endocardium were created using the steps below.

**1.1 Segmentation and Mesh Creation**

A convolutional neural network (CNN) was used to automatically generate a 3D whole-heart multilabel segmentation from the end-diastolic CT time frame [17]. The body of the left atrium (LA), left atrial appendage (LAA) and the left and right pulmonary veins (PVs) were extracted from the multilabel segmentation as a single LA label, as shown in Figure 1A. Segmentations that leaked into the ascending aorta, or where the LAA and left superior PV (LSPV) joined were manually corrected using CemrgApp [18]. Cases where the CNN failed to generate an output were segmented using a semi-automatic region-growing tool and 3D interpolation in CemrgApp.

LA endocardial surface meshes were generated from the LA segmentations using the Medical Image Registration Toolkit, as shown in Figure 1B. Surface meshes were post-processed using CemrgApp. The sleeve extent of the PVs and LAA were labelled semi-automatically using clippers that were generated using a Voronoi diagram representation of the LA. The PVs and mitral valve (MV) were clipped using spherical clippers defined by the user.


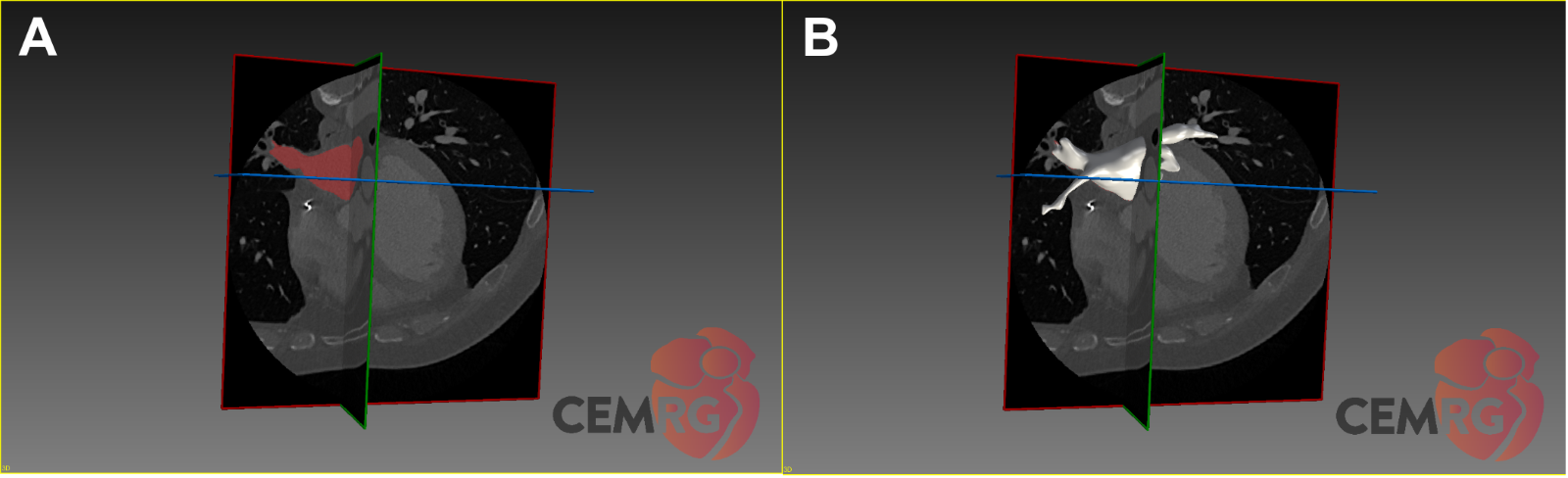


**Figure 1**. Segmentation and surface meshing of the left atrium (LA) from end-diastolic CT image. A: The output segmentation of the LA body, pulmonary veins (PVs) and left atrial appendage (LAA) from a CNN. B: Surface mesh of the LA endocardium extracted from the LA body segmentation.

**1.2 LA Region Definition**

Universal atrial coordinates (UACs) were registered to each patient’s anatomical model by manually selecting 4 landmarks on the endocardium that were used to define the orthogonal axes of the UACs. The landmarks were selected as outlined in Roney et al [21] and were defined as: the LSPV/posterior wall boundary, RSPV/posterior boundary, fossa ovalis and left atrial appendage. The LA body was separated into 5 different regions: posterior wall, septum, lateral wall, anterior wall and inferior wall using the UACs. The pulmonary vein positions were used as landmarks to create the borders between the 5 regions, as depicted in Figure 2A.


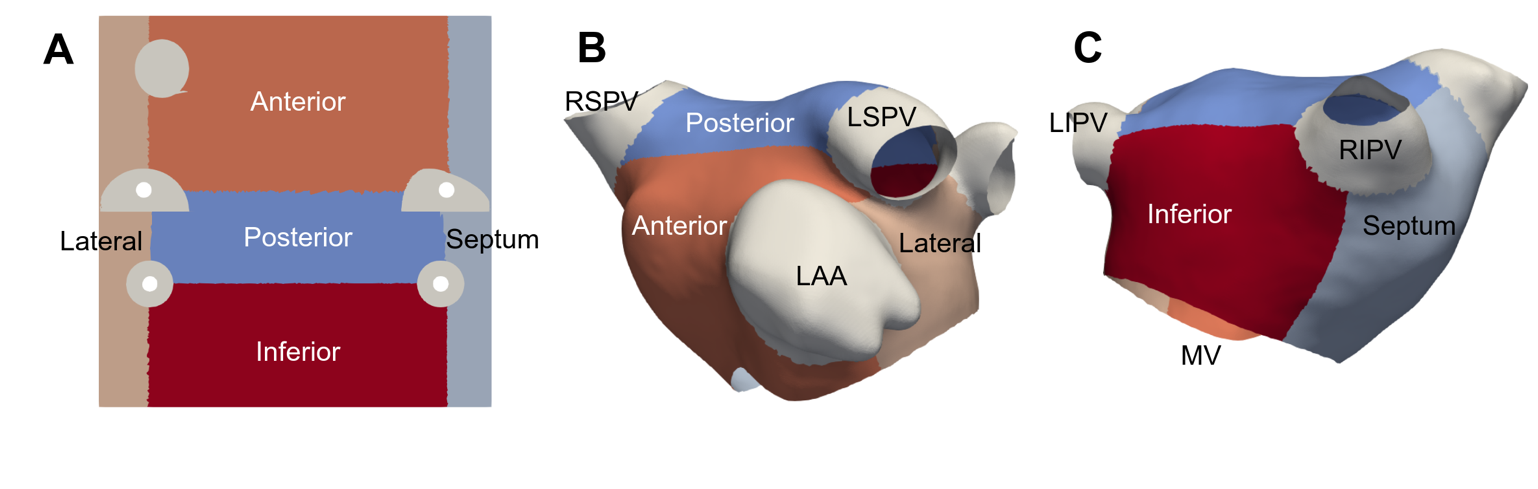


**Figure 2.** Separation of the LA body in 5 regions (posterior, septum, lateral, anterior and inferior walls) using the UACs. **A:** The pulmonary veins (PVs) were used as landmarks to delineate the LA body into 5 regions, denoted by different colors. The PVs and LAA area shaded light gray to show these regions were omitted from the strain calculations. This ensured only strains from the LA body contributed to strain measurement. **B & C:** The 5 regions viewed on the LA anatomy. Abbreviations: LAA – left atrial appendage, LSPV – left superior pulmonary vein, LIPV – left inferior pulmonary vein, RSPV – right superior pulmonary vein, RIPV – right inferior pulmonary vein, MV – mitral valve.

|  | **Inter-observer differences (cm^2^)** |  |
| --- | --- | --- |
| **Region** | **Mean ± Stddev.** | **Mean absolute size (cm^2^)** |
| Posterior | 0.3 ± 1.9 | 21.8 |
| Septum | 1.8 ± 1.5 | 12.1 |
| Lateral | 0.1 ± 0.9 | 6.6 |
| Anterior | 1.4 ± 1.5 | 17.1 |
| Inferior | 0.8 ± 2.1 | 19.1 |

**Table 1:** Inter-observer variability in regional surface area across 10 cases. Models were created by two users independently.

|  | **Inter-observer differences (mm)** |
| --- | --- |
| **Region** | **Mean ± Stddev.** |
| Posterior | 1.5 ± 1.1 |
| Septum | 2.5 ± 1.0 |
| Lateral | 1.8 ± 0.9 |
| Anterior | 2.5 ± 1.3 |
| Inferior | 1.4 ± 0.7 |

**Table 2:** Inter-observer variability in region centre of mass location across 10 cases. Models were created by two users independently.

**2 Feature Tracking**

Three-dimensional LA feature tracking was achieved using the temporal sparse free form deformation method (TSFFD) [40], which simultaneously registers the initial ED frame with all later CT frames. The TSFFD method is based upon the widely used free form deformation method and uses an image similarity matching technique with spatial and temporal regularization that encourages spatially and temporally smooth displacements, which are appropriate for the application of cardiac motion estimation. The TSFFD parameters were previously optimised for the application of LA feature tracking in 10-frame RGCT in [14]. Furthermore, studies show different temporal frame number impacts strain measurements [22]. Therefore, ten RGCT images at 10% increments were used for feature tracking.


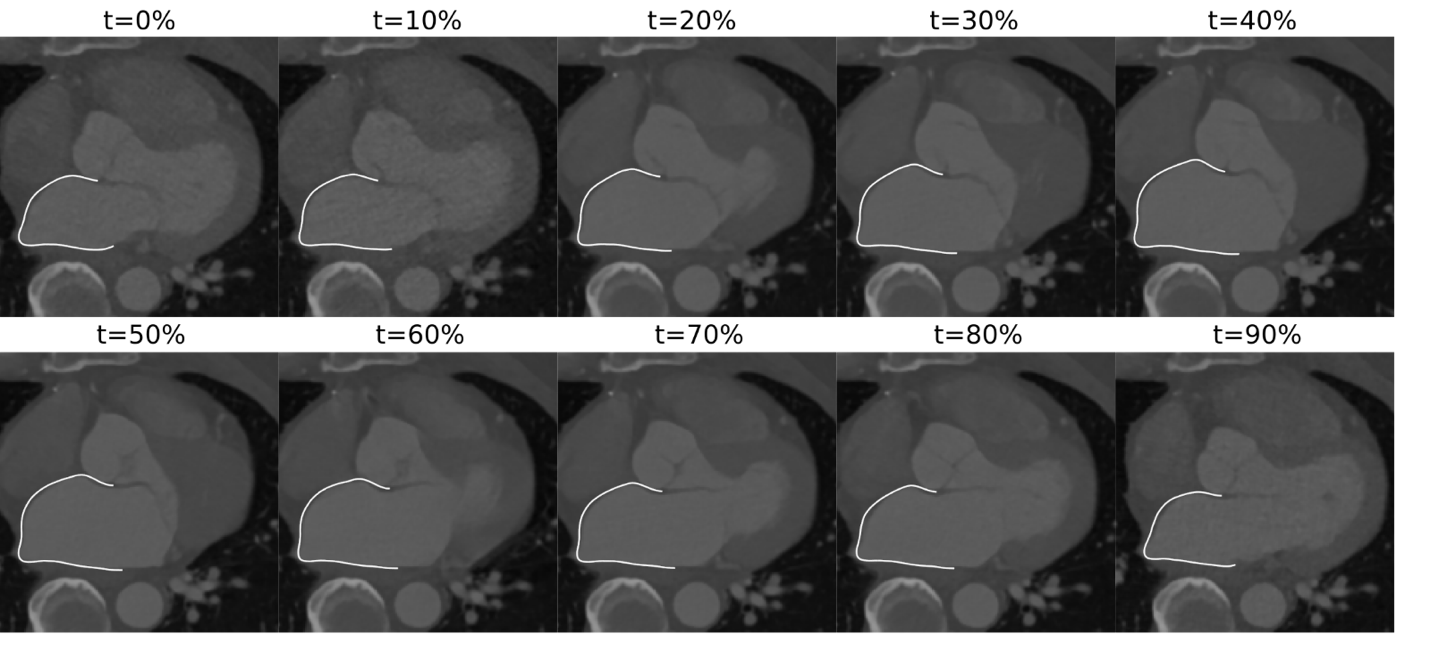
**Figure 3: Three-dimensional LA motion estimation from retrospective gated computed tomography (RGCT) imaging with feature tracking, visualized in the same axial CT slice over multiple temporal frames.** Overlaid LA endocardial surface contours produced by the 3D feature tracking demonstrably track the LA blood pool in the CT frames at 10% increments over the R-R interval.

**3 Strain Measurement**

Deformation in 3D was quantified using area strain, which computes the percentage area change of each local element with respect to the ED model, as shown in equation 1 below. Positive and negative area strain denote expansion and contraction, respectively.


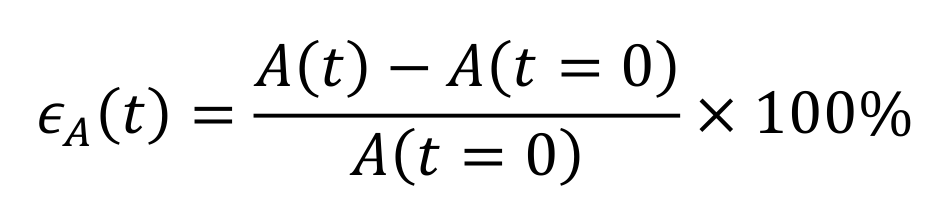


**Equation 1:** Calculation of area strain at time t, which is calculated from the elemental area at time t, A(t), elemental area at end-diastole, A(t=0).

**3.1 Phase Definition**

The three atrial functional phases – reservoir, conduit and contractile – were identified from the global area strain curve and verified using the LA volume curve (Figure 4). Our method to extract phase definitions was determined by the recommendations set out in the consensus document from the EACVI/ASD/Industry Task Force on the Standardization of left atrial, right ventricular, and right atrial deformation imaging using two- dimensional speckle tracking echocardiography (Badano et al 2018) [23], which states that phase definitions may be provided by the LA strain curve. The Task Force recommends that the lowest point of the LA strain curve may be used to define end-diastole, and the onset of atrial contraction can be identified using the sharp downslope following the “horizontal strain curve in diastasis" whilst avoiding the "sharp downslope in the conduit phase at the beginning of diastasis".

Our method involved the following steps. Cubic splines were fit to strain data points, and the inflection point following the maximum strain value was classified as the conduit-contractile phase boundary. All phase separations were reviewed manually. If no appropriate inflection point existed in the strain curve, the inflection point in the volume curve was used to identify the conduit-contractile phase boundary. The conduit-contractile phase boundary was manually checked to be located after the sharp downslope in the conduit phase and following a plateau towards the end of the conduit phase, in line with the Task Force recommendations. The smallest strain value was defined as end-diastole.

Reservoir strain was defined as the maximum minus minimum strain value. Conduit strain was defined as the maximum strain minus the strain value at the conduit-contractile phase boundary, and contractile strain was defined as the reservoir strain minus conduit strain. The strain curve was differentiated to obtain the strain rate (SR) curve, and peak positive SR values within the reservoir phase and peak negative SR values within the conduit and contractile phases established phasic SR measurements. Regional phasic measurements were extracted from the regional strain and SR curves using the global phase definitions.


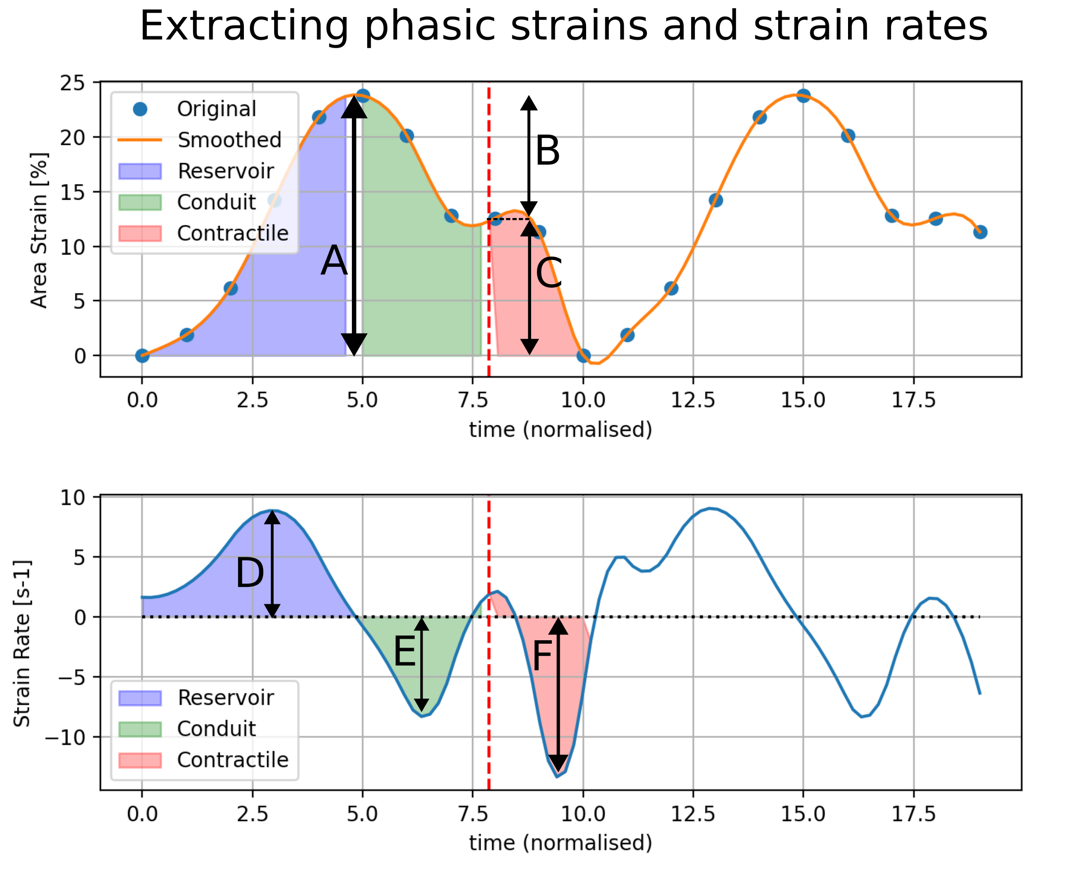


**Figure 4: Definition of the atrial functional phases (reservoir, conduit, contractile) from the global LA body strain curve (top), shown over two beats.** A cubic spline was fitted to the strain data points. The inflection point following peak strain at the end of the reservoir phase in the first beat separated the conduit and contractile phases, shown by the vertical dashed red line. Phase boundaries for all cases were manually reviewed to ensure correct phase definitions. The strain rate curve (bottom) was obtained by taking the first time derivative of the strain curve. Parameters, A: Reservoir strain, B: conduit strain, C: contractile strain, D: reservoir strain rate, E: conduit strain rate, F: contractile strain rate.

**4 CT Scanner Details**

| CT Scanner Model and Manufacturer | Number of Cases | | In-plane resolution [mm] | | Slice thickness [mm] | |
| --- | --- | --- | --- | --- | --- | --- |
| Siemens SOMATOM Definition Flash | 30 | 0.63-0.95 | | 2.0 | |  |
| Siemens SOMATOM Definition AS+ | 9 | 0.32-0.55 | | 0.7 | |  |
| Siemens SOMATOM Definition Edge | 5 | 0.38-0.56 | | 1.0 | |  |
| Siemens SOMATOM Force | 13 | 0.31-0.84 | | 0.7-2.0 | |  |
| GE Medical Systems Discovery CT750 HD | 1 | 0.49 | | 0.8 | |  |
| GE Medical Systems Revolution CT | 11 | 0.39-0.625 | | 0.625 | |  |

**Table 3**: CT Scanner models and manufacturers for the patient cohort.

| Contrast Agent Used | Number of Cases | |
| --- | --- | --- |
| Isovue 300 | 59 |  |
| Isovue 370 | 10 |  |

**Table 4**: Contrast agents used.

|  | Average surface distance [mm] | |  |
| --- | --- | --- | --- |
| Variable (IQR) | Lower quartile | Upper quartile | P-value |
| In-plane resolution (0.42 - 0.77 mm) | 1.1 ± 0.3 | 1.1 ± 0.2 | 0.83 |
| Slice thickness (0.7 – 2 mm) | 1.1 ± 0.3 | 1.1 ± 0.2 | 0.90 |
| Contrast volume usage (81 – 115 ml) | 1.0 ± 0.2 | 1.0 ± 0.2 | 0.74 |
| CT Dose Index volume (16 – 42 mGy) | 1.1 ± 0.2 | 1.1 ± 0.3 | 0.55 |
|  |  |  |  |
| Contrast agent used | Isovue 300 | Isovue 370 |  |
|  | 1.2 ± 0.2 | 1.1 ± 0.2 | 0.22 |

**Table 5**: Differences in the average surface distance (ASD) metric in mm, which quantifies tracking accuracy, with respect to variations in scan settings. Lower ASD values indicate more accurate tracking. ASD values and therefore tracking accuracy were comparable between variations in scan settings.

**
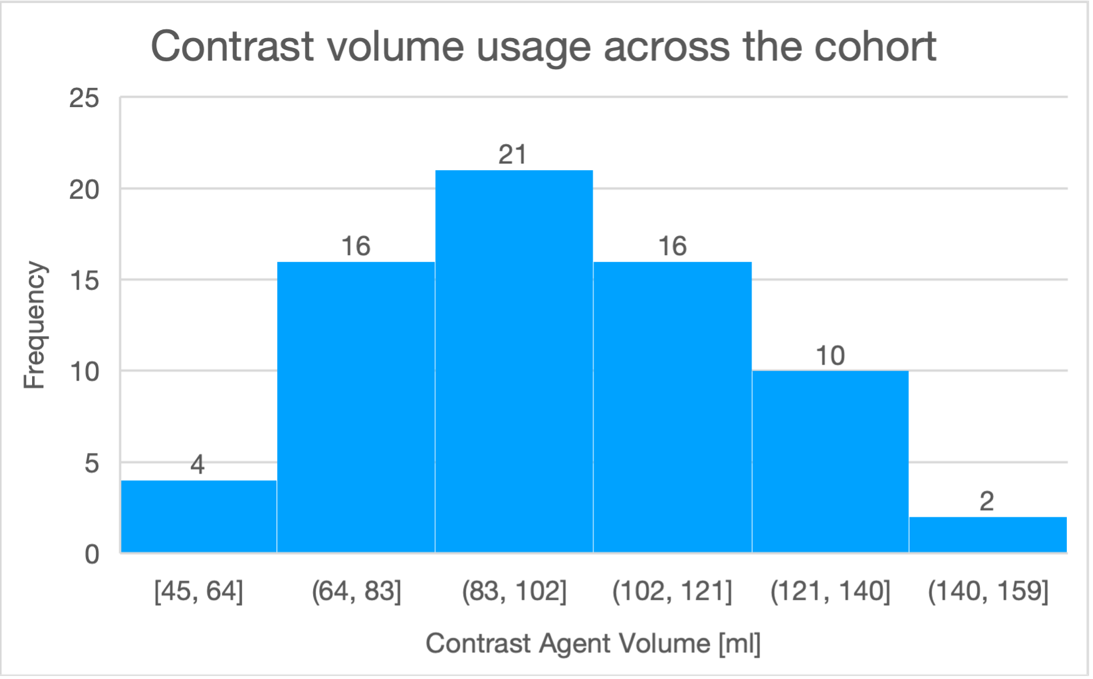
**

**Figure 5**: Contrast volume usage in ml across the cohort.

**
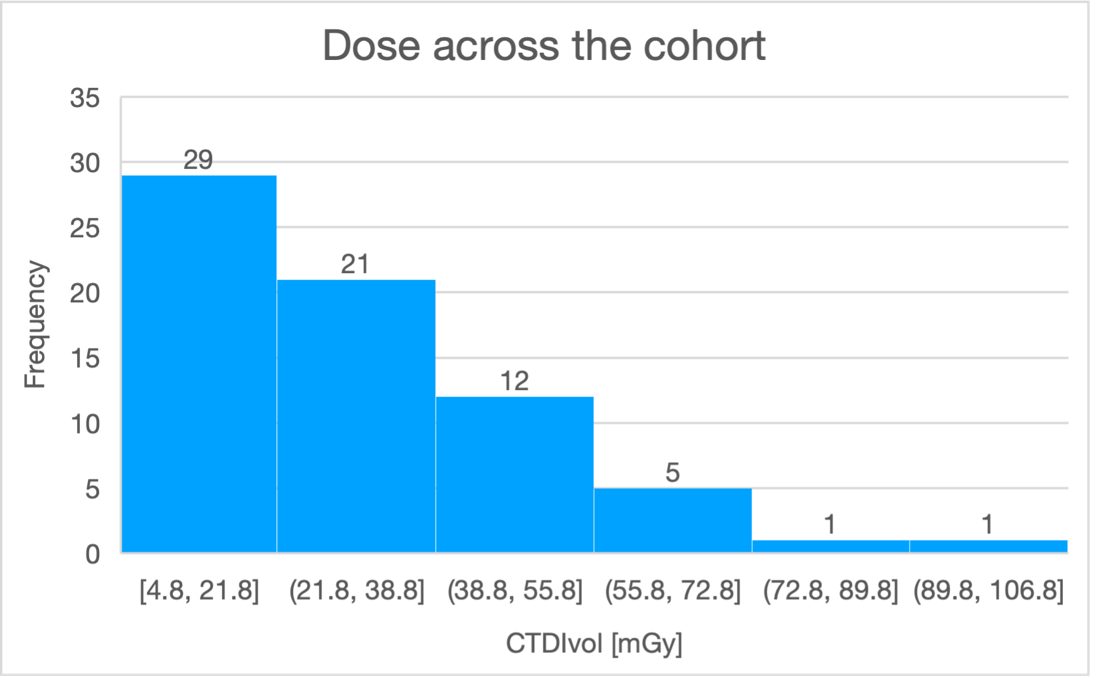
**

**Figure 6**: CT dose index volume (CTDIvol) in mGy across the cohort.

**Results**

**1 Mixed ANOVA Results**

This section details the mixed ANOVA results for testing for group and region interaction.

**1.1 AF Type**

| Variable | Interaction P-value |
| --- | --- |
| **Strain Parameters** |  |
| Reservoir Strain | 0.19 |
| Conduit Strain | 0.21 |
| Contractile Strain | 0.70 |
| **Strain Rate Parameters** |  |
| Reservoir SR | 0.10 |
| Conduit SR | 0.83 |
| Contractile SR | 0.90 |

**Table 6**: Mixed ANOVA results testing for the interaction effect between AF type and region for each strain and strain rate parameter separately.

**1.2 AF Recurrence**

| Variable | Interaction P-value |
| --- | --- |
| **Strain Parameters** |  |
| Reservoir Strain | 0.77 |
| Conduit Strain | 0.32 |
| Contractile Strain | 0.51 |
| **Strain Rate Parameters** |  |
| Reservoir SR | 0.34 |
| Conduit SR | 0.30 |
| Contractile SR | 0.10 |

**Table 7**: Mixed ANOVA results testing for the interaction effect between AF recurrence and region for each strain and strain rate parameter separately.

**2 Differences in Strain and Image Analysis**

**2.1 AF Type**

| **Variable** | **Non-paroxysmal AF (n=21)** | **Paroxysmal AF (n=48)** | **P-value** |
| --- | --- | --- | --- |
| Age, years | 63.9 (10.3) | 59.5 (12.8) | 0.165 |
| Female, n (%) | 8 (38.1) | 19 (39.6) | 1.0 |
| Prior AF Ablation, n (%) | 8 (38.1) | 24 (50.0) | 0.516 |
| Height (cm) | 175.6 (12.6) | 175.0 (12.3) | 0.866 |
| Weight (kg) | 93.1 (26.1) | 89.4 (17.9) | 0.504 |
| BMI | 29.8 (5.8) | 29.2 (5.2) | 0.674 |
| CHADS2-VASc | 2.4 (1.7) | 1.9 (1.5) | 0.208 |
| CHADS2-VASc ≥ 2, n (%) | 14 (66.7) | 28 (58.3) | 0.701 |
| AF recurrence, n (%) | 5.0 (23.8) | 13.0 (27.1) | 1.0 |
| Duration of AF (days) | 1862 (808-4274) | 1716 (690-3037) | 0.221 |
| **Comorbidities** |  |  |  |
| Coronary Artery Disease, n (%) | 4 (19.0) | 6 (12.5) | 0.479 |
| Congestive Heart Failure, n (%) | 3 (14.3) | 7 (14.6) | 1.0 |
| Diabetes Mellitus, n (%) | 6 (28.6) | 5 (10.4) | 0.078 |
| Hypertension, n (%) | 12 (57.1) | 28 (58.3) | 1.0 |
| Hyperlipidemia, n (%) | 8 (38.1) | 21 (43.8) | 0.863 |
| Valvular Disease, n (%) | 3 (14.3) | 1 (2.1) | 0.081 |
| **Ablation Strategy** |  |  |  |
| PVI Ablation Only, n (%) | 11 (52.4) | 28 (58.3) | 0.845 |
| Extra-PV Ablation Only, n (%) | 3 (14.3) | 5 (10.4) | 0.692 |
| PVI and Extra-PV Ablation, n (%) | 7 (33.3) | 15 (31.3) | 1.0 |

**Table 8**: Patient characteristics separated by non-paroxysmal and paroxysmal AF.

| **Variable** | **Non-paroxysmal AF (n=21)** | **Paroxysmal AF (n=48)** | **P-value** |
| --- | --- | --- | --- |
| **CT LA Parameters** |  |  |  |
| LA EDV [ml] | 109.7 (46.4) | 80.8 (35.2) | **0.006** |
| LA ESV [ml] | 144.9 (41.1) | 126.1 (39.4) | 0.077 |
| LA EDVI [ml/m^2^] | 54.4 (26.0) | 39.7 (17.0) | **0.007** |
| LA ESVI [ml/m^2^] | 70.9 (22.8) | 61.9 (18.7) | 0.088 |
| LA SV [ml] | 41.3 (19.6) | 49.5 (18.2) | 0.097 |
| LAEF [%] | 29.6 (15.7) | 39.1 (12.8) | **0.011** |
| **CT LV Parameters** |  |  |  |
| LV EDV [ml] | 188.7 (51.9) | 181.5 (32.1) | 0.483 |
| LV ESV [ml] | 96.2 (33.2) | 90.5 (22.3) | 0.405 |
| LV EDVI [ml/m^2^] | 90.5 (18.2) | 88.7 (11.6) | 0.63 |
| LV ESVI [ml/m^2^] | 46.1 (13.2) | 44.1 (9.2) | 0.482 |
| LV SV [ml] | 92.5 (24.8) | 91.0 (19.0) | 0.783 |
| LVEF [%] | 49.4 (6.8) | 50.3 (6.8) | 0.6 |

**Table 9: Comparison of CT volumetric parameters between paroxysmal and non-paroxysmal AF.** Abbreviations: LA- left atrium, LV – left ventricle, EDV – end-diastolic volume, ESV – end-systolic volume, EDVI – end-diastolic volume index, ESVI – end-systolic volume index, SV – stroke volume, LAEF – left atrial emptying fraction, LVEF – left ventricular ejection fraction.

**
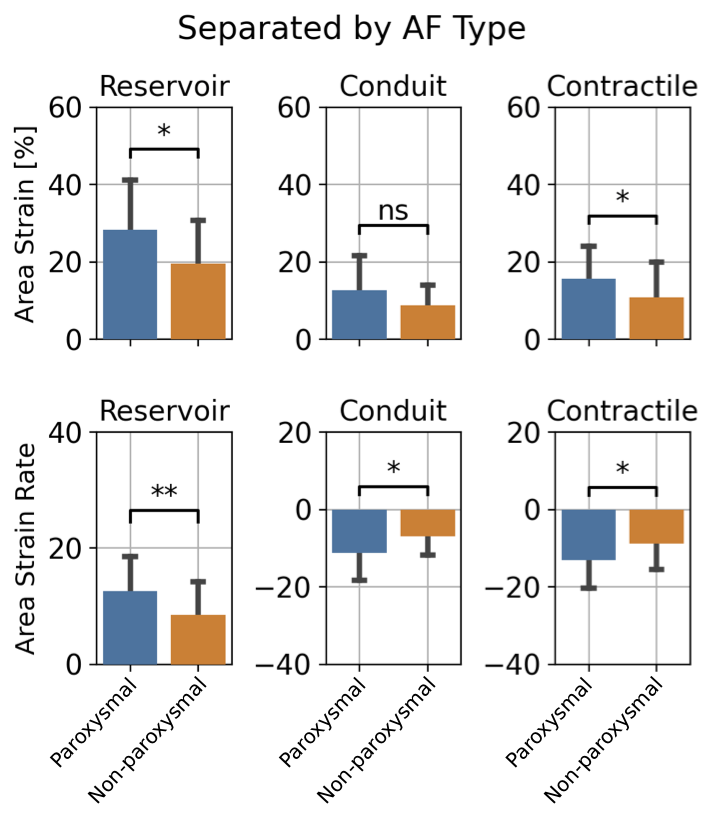
**

**Figure 7: Global Comparison of the global phasic strains and peak strain rates from 3D LA motion between paroxysmal and non-paroxysmal AF groups.** Peak reservoir strain rate, reflecting passive mechanical function, displayed the most significant differences in patients with more persistent AF. P-value legend, ns: not significant; *: P < 0.05, **: P<0.01.

**
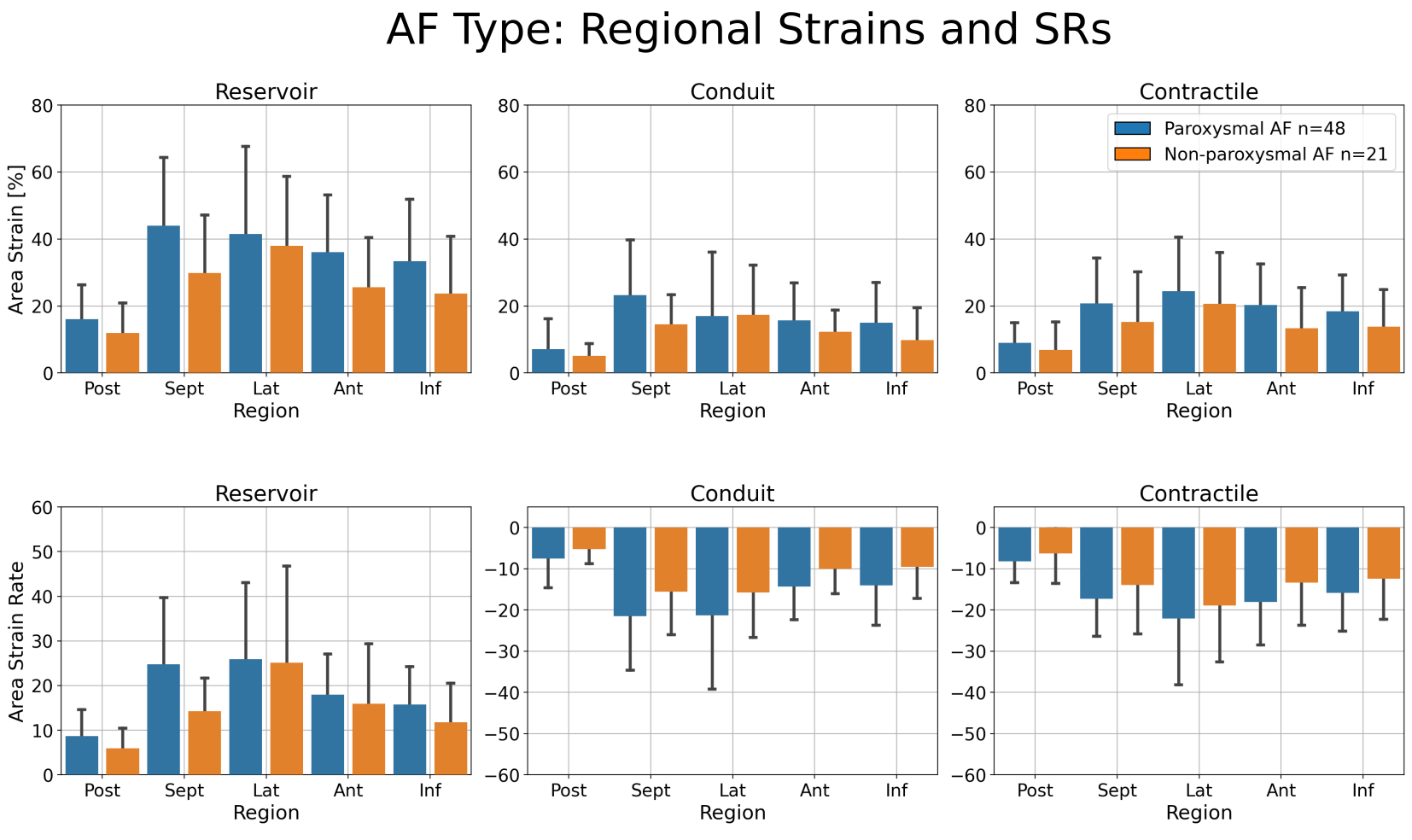
**

**Figure 8**: Regional reservoir and contractile strain (top row) and regional reservoir, conduit and contractile SR (bottom row), separated by AF type.

**2.2 AF Recurrence**

**
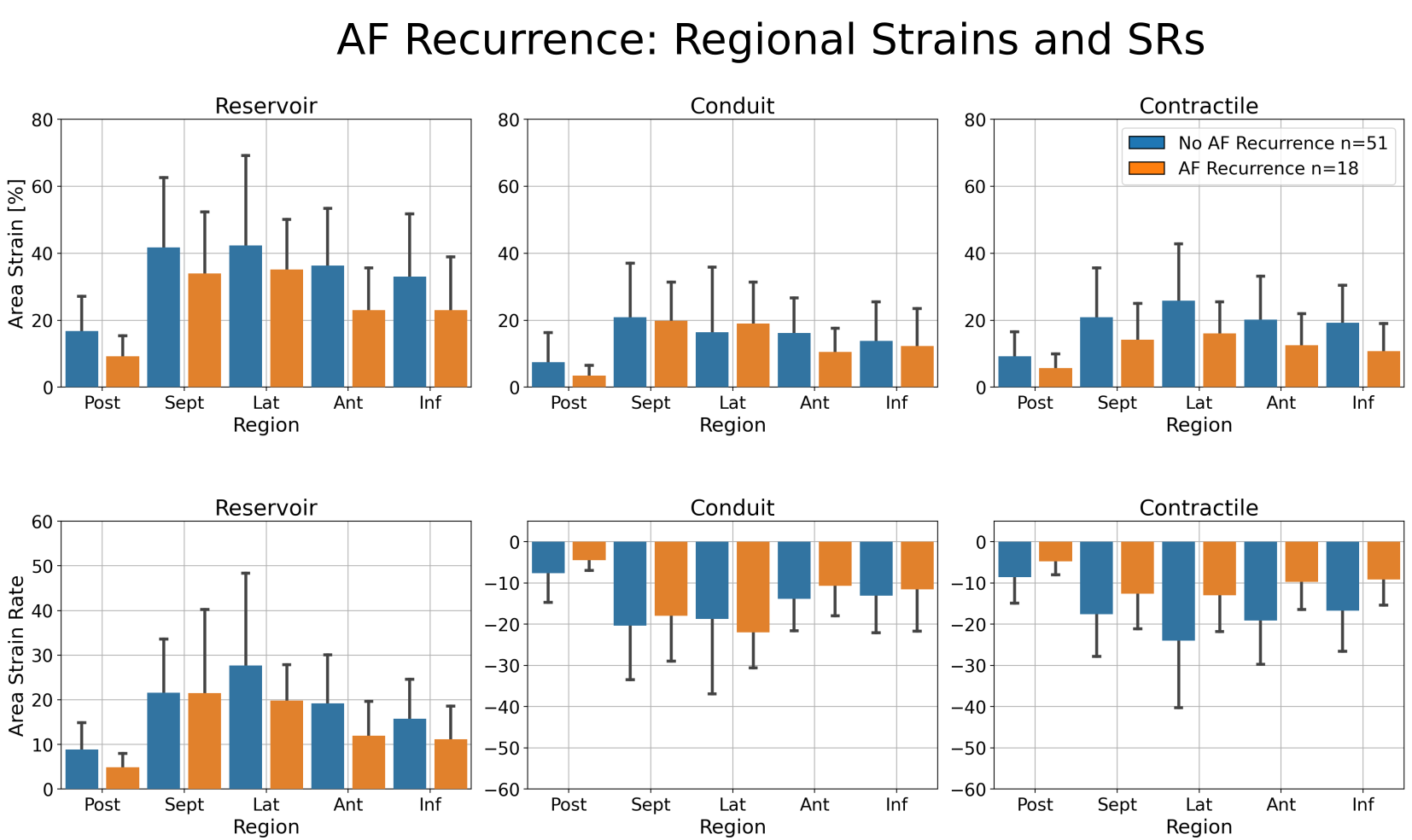
**

**Figure 9**: Regional reservoir and contractile strain (top row) and regional reservoir, conduit and contractile SR (bottom row), separated by recurrent AF.

**3 Receiver Operating Characteristic Analysis**

**3.1 AF Type**

| Variable | Mean AUC [95% CI] | P-value |
| --- | --- | --- |
| **Reservoir Area Strains** |  |  |
| Global | 0.67 [0.52-0.8] | 0.011 |
| Posterior | 0.64 [0.5-0.79] | 0.019 |
| Septum | 0.75 [0.62-0.86] | 0.001 |
| Lateral | 0.5 [0.34-0.66] | 0.099 |
| Anterior | 0.67 [0.53-0.8] | 0.008 |
| Inferior | 0.67 [0.51-0.8] | 0.014 |
| **Conduit Area Strains** |  |  |
| Global | 0.65 [0.5-0.78] | 0.014 |
| Posterior | 0.53 [0.39-0.66] | 0.037 |
| Septum | 0.69 [0.55-0.82] | 0.007 |
| Lateral | 0.47 [0.31-0.63] | 0.121 |
| Anterior | 0.58 [0.43-0.72] | 0.034 |
| Inferior | 0.66 [0.51-0.79] | 0.012 |
| **Contractile Area Strains** |  |  |
| Global | 0.67 [0.52-0.8] | 0.011 |
| Posterior | 0.66 [0.5-0.81] | 0.024 |
| Septum | 0.66 [0.52-0.8] | 0.012 |
| Lateral | 0.57 [0.39-0.73] | 0.076 |
| Anterior | 0.68 [0.52-0.81] | 0.013 |
| Inferior | 0.62 [0.46-0.76] | 0.028 |
| **Reservoir Peak Strain Rates** |  |  |
| Global | 0.71 [0.57-0.83] | 0.004 |
| Posterior | 0.67 [0.52-0.81] | 0.014 |
| Septum | 0.75 [0.62-0.87] | 0.001 |
| Lateral | 0.58 [0.42-0.74] | 0.055 |
| Anterior | 0.61 [0.46-0.75] | 0.026 |
| Inferior | 0.65 [0.49-0.78] | 0.017 |
| **Conduit Peak Strain Rates** |  |  |
| Global | 0.74 [0.59-0.87] | 0.005 |
| Posterior | 0.6 [0.45-0.74] | 0.028 |
| Septum | 0.64 [0.5-0.79] | 0.019 |
| Lateral | 0.6 [0.45-0.76] | 0.040 |
| Anterior | 0.66 [0.52-0.79] | 0.009 |
| Inferior | 0.65 [0.48-0.8] | 0.031 |
| **Contractile Peak Strain Rates** |  |  |
| Global | 0.67 [0.52-0.8] | 0.011 |
| Posterior | 0.65 [0.51-0.81] | 0.022 |
| Septum | 0.64 [0.48-0.79] | 0.029 |
| Lateral | 0.55 [0.38-0.71] | 0.078 |
| Anterior | 0.65 [0.5-0.78] | 0.014 |
| Inferior | 0.6 [0.43-0.75] | 0.047 |
| **Additional Parameters** |  |  |
| CT LA EDV [ml] | 0.71 [0.55-0.84] | 0.009 |
| CT LA EDVI [ml/m^2^] | 0.68 [0.52-0.82] | 0.016 |
| CT LAEF [%] | 0.68 [0.53-0.81] | 0.010 |
| CHADS2-VASc | 0.59 [0.43-0.73] | 0.037 |

**Table 10**: Mean area-under-curve (AUC) and 95% confidence interval values from ROC analysis investigating association with non-paroxysmal AF.

**3.2 AF Recurrence**

| Variable | Mean AUC [95% CI] | P-value |
| --- | --- | --- |
| **Reservoir Area Strains** |  |  |
| Global | 0.71 [0.57-0.84] | 0.005 |
| Posterior | 0.75 [0.61-0.88] | 0.003 |
| Septum | 0.61 [0.45-0.77] | 0.043 |
| Lateral | 0.56 [0.43-0.69] | 0.022 |
| Anterior | 0.74 [0.61-0.86] | 0.002 |
| Inferior | 0.68 [0.53-0.82] | 0.013 |
| **Conduit Area Strains** |  |  |
| Global | 0.58 [0.43-0.73] | 0.040 |
| Posterior | 0.69 [0.54-0.83] | 0.012 |
| Septum | 0.5 [0.33-0.69] | 0.143 |
| Lateral | 0.36 [0.21-0.51] | 0.208 |
| Anterior | 0.68 [0.53-0.82] | 0.013 |
| Inferior | 0.54 [0.38-0.7] | 0.074 |
| **Contractile Area Strains** |  |  |
| Global | 0.72 [0.58-0.84] | 0.003 |
| Posterior | 0.65 [0.51-0.78] | 0.011 |
| Septum | 0.65 [0.5-0.79] | 0.017 |
| Lateral | 0.67 [0.53-0.79] | 0.006 |
| Anterior | 0.67 [0.53-0.81] | 0.011 |
| Inferior | 0.72 [0.58-0.85] | 0.005 |
| **Reservoir Peak Strain Rates** |  |  |
| Global | 0.65 [0.5-0.79] | 0.017 |
| Posterior | 0.75 [0.6-0.87] | 0.003 |
| Septum | 0.56 [0.37-0.73] | 0.100 |
| Lateral | 0.59 [0.45-0.74] | 0.031 |
| Anterior | 0.75 [0.61-0.89] | 0.005 |
| Inferior | 0.67 [0.53-0.82] | 0.014 |
| **Conduit Peak Strain Rates** |  |  |
| Global | 0.62 [0.45-0.77] | 0.040 |
| Posterior | 0.69 [0.54-0.82] | 0.009 |
| Septum | 0.57 [0.38-0.74] | 0.094 |
| Lateral | 0.32 [0.19-0.45] | 0.196 |
| Anterior | 0.64 [0.47-0.79] | 0.034 |
| Inferior | 0.58 [0.42-0.74] | 0.055 |
| **Contractile Peak Strain Rate** |  |  |
| Global | 0.74 [0.61-0.86] | 0.002 |
| Posterior | 0.69 [0.53-0.82] | 0.012 |
| Septum | 0.64 [0.48-0.78] | 0.024 |
| Lateral | 0.71 [0.58-0.82] | 0.002 |
| Anterior | 0.77 [0.65-0.89] | 0.001 |
| Inferior | 0.74 [0.6-0.86] | 0.003 |
| **Additional Parameters** |  |  |
| CT LA EDV [ml] | 0.68 [0.51-0.85] | 0.034 |
| CT LA EDVI [ml/m^2^] | 0.68 [0.51-0.85] | 0.034 |
| CT LAEF [%] | 0.68 [0.53-0.81] | 0.016 |
| CHADS2-VASc | 0.63 [0.47-0.78] | 0.029 |

**Table 11**: Mean area-under-curve (AUC) and 95% confidence interval values from ROC analysis investigating association with AF recurrence post-ablation.

**4 Unsupervised Machine Learning Clustering**


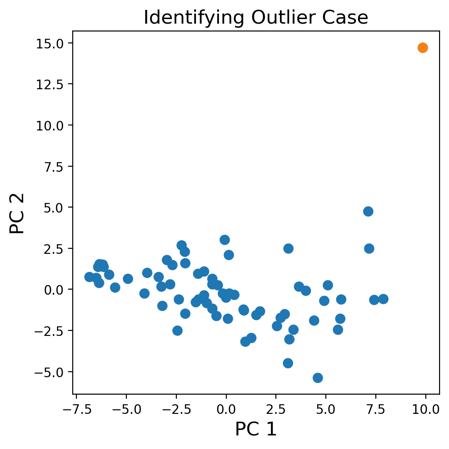


**Figure 10: Initial K-Means Clustering results revealed an outlier case (top right data point in orange).** The outlier case was removed before clustering analysis was repeated.


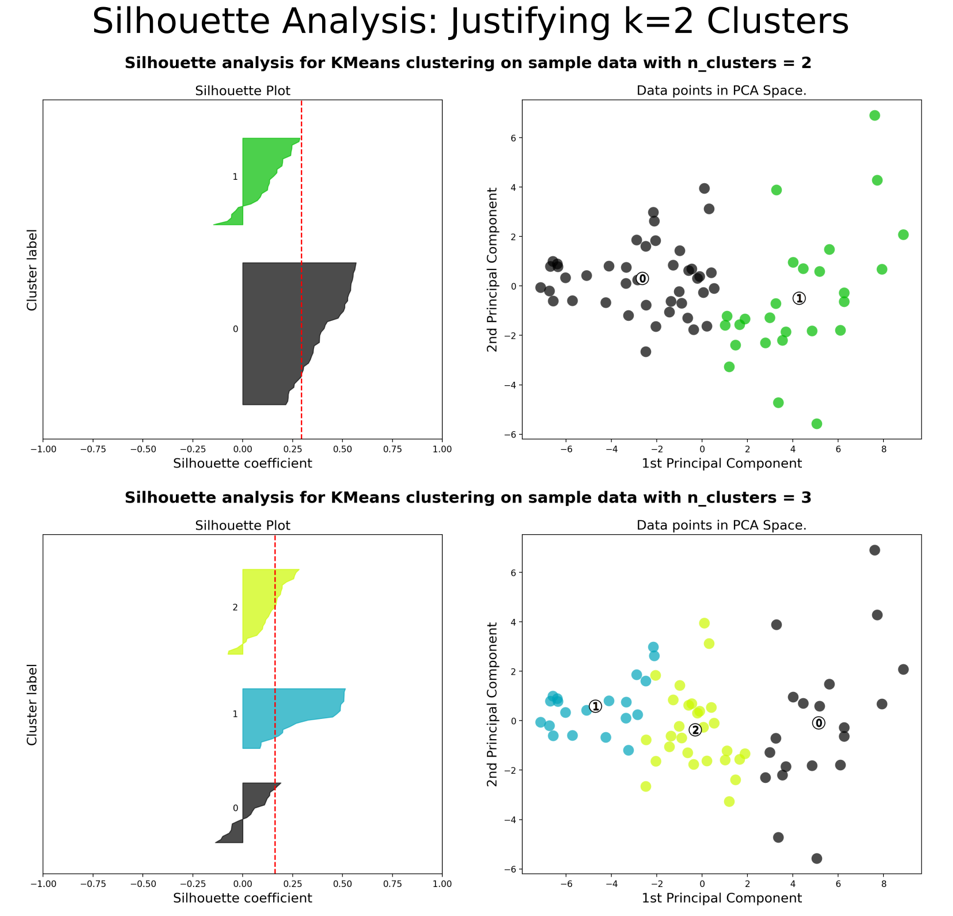


**Figure 11: Silhouette analysis plot (left) and data points** **in space defined by first two principal components (right) using K-Means clustering for k=2 and 3 clusters**. The red vertical line denotes the mean silhouette score across all data points. A higher mean silhouette score and silhouette coefficients within each cluster was observed when using k=2 clusters.

| Variable | Low strain  Cluster N=42 | High strain Cluster N=26 | P-value |
| --- | --- | --- | --- |
| Age | 63.22 (11.0) | 56.78 (13.4) | * |
| Female Sex, n (%) | 21 (50.0) | 5 (19.2) | * |
| Prior AF Ablation, n (%) | 25 (59.5) | 7 (26.9) | * |
| BMI | 29.08 (5.8) | 29.75 (4.8) | ns |
| CHADS2-VASc | 2.31 (1.5) | 1.62 (1.6) | ns |
| CHADS2-VASc ≥ 2, n (%) | 28 (66.7) | 13 (50.0) | ns |
| Paroxysmal AF, n (%) | 26 (61.9) | 21 (81.0) | ns |
| Persistent AF, n (%) | 8 (19.0) | 3 (11.5) | ns |
| Long-standing persistent AF, n (%) | 8 (19.1) | 2 (7.7) | ns |
| AF Recurrence, n (%) | 16 (38.1) | 2 (7.7) | * |
| **Comorbidities** |  |  |  |
| Coronary Artery Disease, n (%) | 7 (16.7) | 3 (11.5) | ns |
| Congestive Heart Failure, n (%) | 5 (11.9) | 4 (15.4) | ns |
| Diabetes Mellitus, n (%) | 9 (21.43) | 2 (7.7) | ns |
| Hypertension, n (%) | 26 (61.9) | 13 (50.0) | ns |
| Hyperlipidemia, n (%) | 18 (42.9) | 10 (38.5) | ns |
| Obstructive Sleep Apnea, n (%) | 13 (31.0) | 10 (38.5) | ns |
| Valvular Disease, n (%) | 3 (7.14) | 0 (0.0) | ns |
| **Ablation Strategy** |  |  |  |
| PVI Ablation Only | 23 (54.8) | 14 (53.8) | ns |
| PVI and Extra-PV Ablation | 14 (33.3) | 12 (46.2) | ns |
| Extra-PV Ablation Only | 5 (11.9) | 0 (0.0) | ns |
| **CT-Derived** **Parameters** |  |  |  |
| CT LVEF [%] | 49.39 (6.0) | 51.92 (6.4) | ns |
| CT LAEF [%] | 29.71 (12.5) | 47.44 (9.3) | *** |
| CT LA EDV [ml] | 102.32 (43.7) | 67.43 (23.7) | *** |
| CT LA ESV [ml] | 136.12 (46.5) | 124.0 (28.7) | ns |
| Global Reservoir Strain [%] | 17.46 (7.5) | 37.68 (8.7) | *** |
| Global Conduit Strain [%] | 8.6 (4.1) | 14.61 (8.3) | *** |
| Global Contractile Strain [%] | 8.86 (5.6) | 23.07 (5.1) | *** |
| Global Reservoir SR | 7.67 (3.2) | 16.99 (5.2) | *** |
| Global Conduit SR | -7.3 (3.9) | -12.86 (5.5) | *** |
| Global Contractile SR | -7.34 (4.6) | -19.28 (4.4) | *** |

**Table 12**: Cluster characteristics. P-value legend, ns: not significant; *: P < 0.05, **: P < 0.01, ***: P < 0.001.

**5 Secondary Analysis: Prior Ablation**

| Variable | Prior Ablation (n=32) | First-time Ablation (n=37) | P-value |
| --- | --- | --- | --- |
| Age, years | 59.4 (11.1) | 62.0 (13.1) | 0.371 |
| Female, n (%) | 14.0 (43.8) | 13.0 (35.1) | 0.628 |
| Height (cm) | 177.0 (13.0) | 173.6 (11.6) | 0.252 |
| Weight (kg) | 90.1 (22.2) | 90.9 (19.4) | 0.868 |
| BMI | 28.6 (5.4) | 30.1 (5.3) | 0.239 |
| CHADS2-VASc | 1.8 (1.5) | 2.4 (1.6) | 0.108 |
| CHADS2-VASc ≥ 2, n (%) | 15.0 (46.9) | 27.0 (73.0) | **0.049** |
| Paroxysmal AF, n (%) | 24.0 (75.0) | 24.0 (64.9) | 0.516 |
| Non-paroxysmal AF, n (%) | 8 (25.0) | 13 (35.1) | 0.516 |
| AF recurrence, n (%) | 10.0 (31.2) | 8.0 (21.6) | 0.526 |
| Durations of AF (days) | 2564 (1087-4002) | 1328 (454-2563) | **0.023** |
| **Comorbidities** |  |  |  |
| Coronary Artery Disease, n (%) | 2.0 (6.2) | 8.0 (21.6) | 0.143 |
| Congestive Heart Failure, n (%) | 2.0 (6.2) | 8.0 (21.6) | 0.143 |
| Diabetes Mellitus, n (%) | 6.0 (18.8) | 5.0 (13.5) | 0.793 |
| Hypertension, n (%) | 16.0 (50.0) | 24.0 (64.9) | 0.316 |
| Hyperlipidemia, n (%) | 13.0 (40.6) | 16.0 (43.2) | 1.0 |
| Valvular Disease, n (%) | 1.0 (3.1) | 3.0 (8.1) | 0.714 |
| **Ablation Strategy** |  |  |  |
| PVI Ablation Only, n (%) | 16 (50.0) | 23 (62.2) | 0.440 |
| PVI and Extra-PV Ablation, n (%) | 11 (34.4) | 11 (29.7) | 0.878 |
| Extra-PV Ablation Only, n (%) | 5 (15.6) | 3 (8.1) | 0.457 |
|  |  |  |  |
|  |  |  |  |

**Table 13**: Demographics of study cohort separated by first-time and prior ablation.

| Variable | | Prior AF ablation (n=32) | No Prior AF ablation (n=37) | P-value |
| --- | --- | --- | --- | --- |
| **LA Parameters** |  | |  |  |
| LA EDV [ml] | 91.0 (36.5) | | 88.4 (44.7) | 0.793 |
| LA ESV [ml] | 130.4 (39.0) | | 133.1 (42.4) | 0.782 |
| LA EDVI [ml/m2] | 45.1 (21.6) | | 43.3 (20.9) | 0.739 |
| LA ESVI [ml/m2] | 63.8 (20.9) | | 65.4 (19.9) | 0.748 |
| LA SV [ml] | 43.7 (19.2) | | 49.9 (18.4) | 0.18 |
| LAEF [%] | 33.4 (13.3) | | 38.6 (14.9) | 0.137 |
| **LV Parameters** |  | |  |  |
| LV EDV [ml] | 181.3 (37.2) | | 185.7 (40.7) | 0.646 |
| LV ESV [ml] | 90.7 (22.1) | | 93.5 (29.2) | 0.667 |
| LA EDVI [ml/m2] | 87.7 (12.8) | | 90.6 (14.6) | 0.385 |
| LA ESVI [ml/m2] | 44.0 (9.8) | | 45.4 (11.2) | 0.596 |
| LV SV [ml] | 90.6 (20.2) | | 92.2 (21.5) | 0.748 |
| LVEF [%] | 50.1 (5.8) | | 50.0 (7.6) | 0.968 |

**Table 14**: CT volumetric analysis separated by first-time and prior ablation.

**5.1 Mixed ANOVA Results: Prior Ablation Groups and Region Interaction**

| Variables | Interaction P-value |
| --- | --- |
| **Strain Parameters** |  |
| Reservoir Strain | 0.44 |
| Conduit Strain | 0.97 |
| Contractile Strain | 0.13 |
| **Strain Parameters** |  |
| Reservoir SR | 0.38 |
| Conduit SR | 0.98 |
| Contractile SR | 0.29 |

**Table 15**: Mixed ANOVA results for the interaction effect between prior ablation and region for each strain and SR parameter separately.

**5.2 Global and Regional Strain Differences**


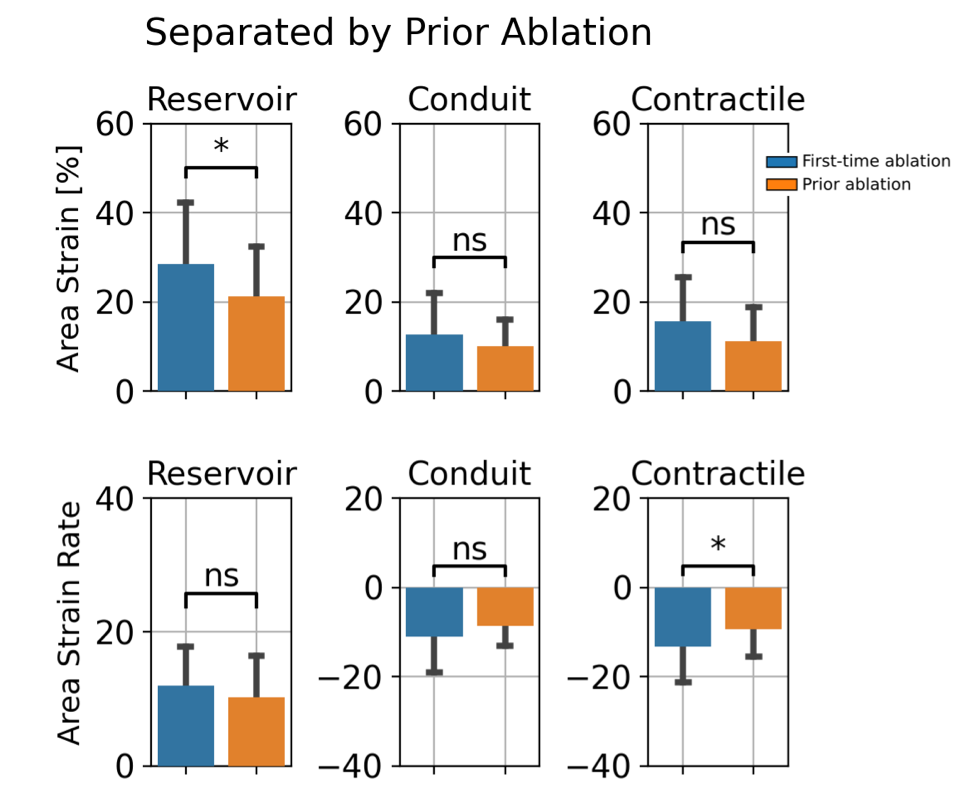


**Figure 12**: Comparison of the global phasic strains and peak phasic strain rates between first-time and prior ablation groups. P-value legend, ns: not significant; *: P < 0.05.


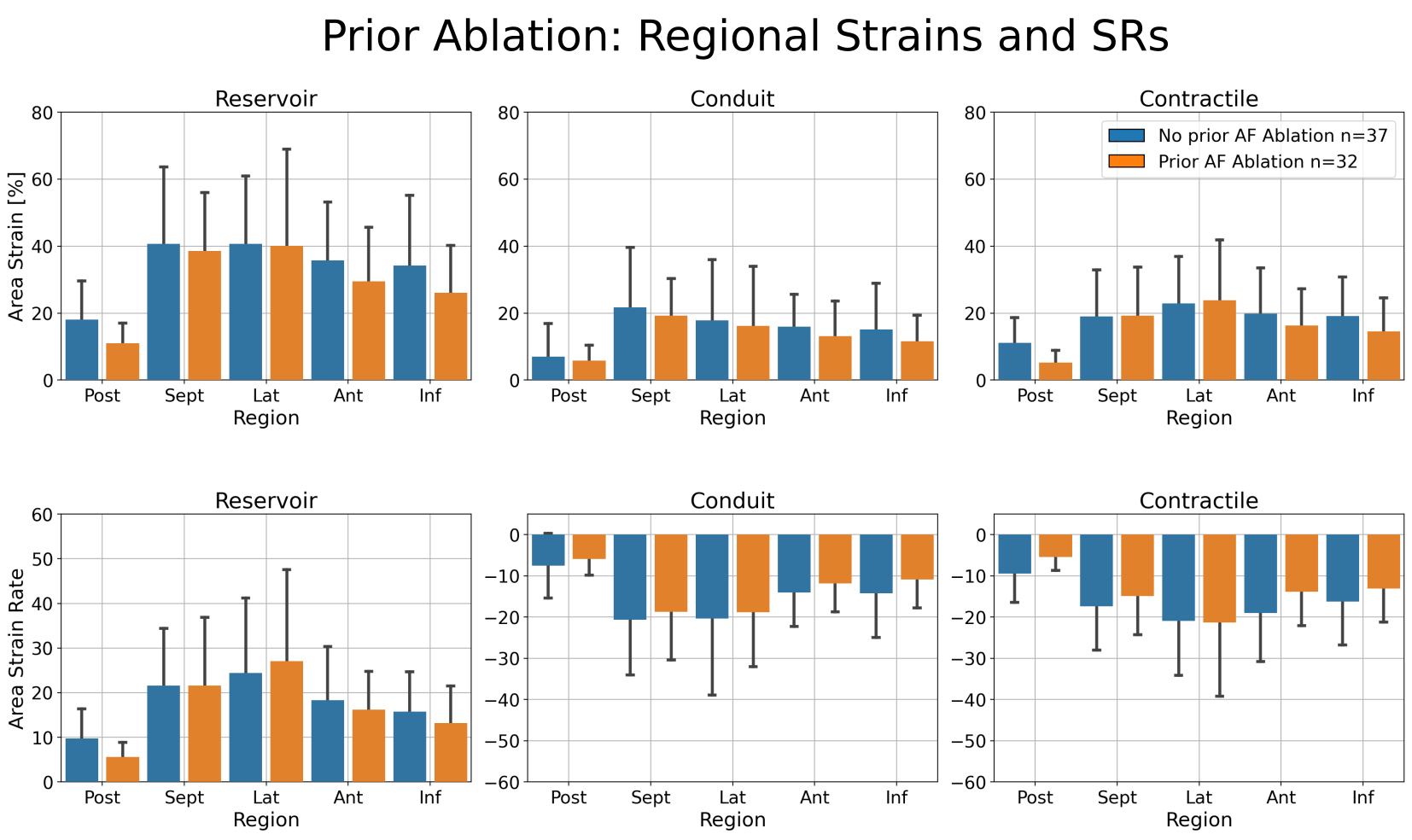


**Figure 13**: Regional reservoir and contractile strain (top row) and regional reservoir, conduit and contractile SR (bottom row), separated by first-time vs prior ablation.

**5.3 ROC Analysis**

| Variable | AUC [95% CI] | P-value |
| --- | --- | --- |
| **Reservoir Area Strains** |  |  |
| Global | 0.65 [0.5-0.77] | 0.011 |
| Posterior | 0.69 [0.57-0.81] | 0.002 |
| Septum | 0.51 [0.37-0.65] | 0.054 |
| Lateral | 0.58 [0.43-0.72] | 0.034 |
| Anterior | 0.61 [0.47-0.75] | 0.021 |
| Inferior | 0.59 [0.45-0.72] | 0.020 |
| **Conduit Area Strains** |  |  |
| Global | 0.58 [0.44-0.71] | 0.023 |
| Posterior | 0.5 [0.37-0.64] | 0.050 |
| Septum | 0.52 [0.38-0.65] | 0.041 |
| Lateral | 0.54 [0.39-0.67] | 0.041 |
| Anterior | 0.6 [0.45-0.73] | 0.023 |
| Inferior | 0.54 [0.41-0.68] | 0.034 |
| **Contractile Area Strains** |  |  |
| Global | 0.63 [0.49-0.76] | 0.013 |
| Posterior | 0.74 [0.61-0.85] | 0.001 |
| Septum | 0.5 [0.37-0.64] | 0.050 |
| Lateral | 0.51 [0.38-0.66] | 0.054 |
| Anterior | 0.57 [0.42-0.72] | 0.044 |
| Inferior | 0.61 [0.47-0.74] | 0.017 |
| **Reservoir Peak Strain Rates** |  |  |
| Global | 0.6 [0.46-0.74] | 0.023 |
| Posterior | 0.72 [0.6-0.84] | 0.001 |
| Septum | 0.51 [0.37-0.65] | 0.054 |
| Lateral | 0.5 [0.36-0.64] | 0.059 |
| Anterior | 0.55 [0.42-0.7] | 0.037 |
| Inferior | 0.58 [0.44-0.71] | 0.023 |
| **Conduit Peak Strain Rates** |  |  |
| Global | 0.56 [0.42-0.69] | 0.028 |
| Posterior | 0.56 [0.43-0.71] | 0.034 |
| Septum | 0.53 [0.39-0.67] | 0.045 |
| Lateral | 0.51 [0.38-0.65] | 0.045 |
| Anterior | 0.57 [0.43-0.71] | 0.031 |
| Inferior | 0.58 [0.45-0.72] | 0.023 |
| **Contractile Peak Strain Rates** |  |  |
| Global | 0.64 [0.5-0.77] | 0.012 |
| Posterior | 0.67 [0.54-0.8] | 0.006 |
| Septum | 0.56 [0.42-0.69] | 0.028 |
| Lateral | 0.53 [0.39-0.68] | 0.053 |
| Anterior | 0.63 [0.5-0.75] | 0.008 |
| Inferior | 0.58 [0.44-0.7] | 0.018 |
| **Additional Parameters** |  |  |
| CT LA EDV [ml] | 0.54 [0.4-0.68] | 0.041 |
| CT LA EDVI [ml/m^2^] | 0.54 [0.39-0.68] | 0.048 |
| CT LAEF [%] | 0.61 [0.48-0.74] | 0.013 |
| CHADS2-VASc | 0.61 [0.48-0.75] | 0.017 |

**Table 16:** Mean area-under-curve (AUC) and 95% confidence interval values from ROC analysis investigating association with prior ablation.

**5.3.3 DeLong Analysis for Prior Ablation Classification**

| Posterior Wall vs Corresponding Global Variable | P-value |
| --- | --- |
| **Strains** |  |
| Reservoir | 0.28 |
| Conduit | 0.31 |
| Contractile | **0.01** |
| **Strain Rates** |  |
| Reservoir | **0.03** |
| Conduit | 0.93 |
| Contractile | 0.38 |

**Table 17: Posterior wall vs corresponding global strain parameters.** Posterior wall contractile strain and reservoir SR parameters gave significantly improved ROC curves compared to the corresponding global parameters for predicting prior ablation. P-values between ROC curves were calculated using DeLong’s Method.

**6 Sample Size Power Analysis**

| Subgroup | Number of patients | | Differences powered to detect | | Observed differences | |
| --- | --- | --- | --- | --- | --- | --- |
|  | Recurrence | Freedom | Reservoir | Contractile | Reservoir | Contractile |
| Paroxysmal AF | 13 | 35 | 9.1% | 6.4% | 9.0% | 5.9% |
| Non-paroxysmal AF | 5 | 16 | 14.5% | 9.9% | 8.2% | 7.2% |
| All | 18 | 51 | 7.7% | 5.4% | 8.5% | 6.6% |

**Table 18: Power calculation results.** Power of 80% and an alpha level of 0.05 were used. Standard deviations of 10% and 7% were used for the reservoir and contractile parameters, respectively, as informed from prior 3D RGCT-derived LA strains in heart failure patients (Sillett et al 2024) and typical ratios between reservoir and contractile strains (Hopman et al 2023). AF type subgroup analyses was not sufficiently powered to detect the observed differences between recurrence and freedom of AF cases.

**7. Group Overlap Analysis**

**7.1 2x2 ANOVA**

| **Dependent variable** | **Independent variables** | **sum_sq** | **df** | **F-statistic** | **P-value** |
| --- | --- | --- | --- | --- | --- |
| Reservoir Strain | Recurrence | 1042.01 | 1.0 | 7.209 | 0.009 |
|  | Paroxysmal AF | 1159.58 | 1.0 | 8.022 | 0.006 |
|  | Interaction | 2.61 | 1.0 | 0.018 | 0.893 |
| Conduit Strain | Recurrence | 58.97 | 1.0 | 0.917 | 0.342 |
|  | Paroxysmal AF | 233.56 | 1.0 | 3.632 | 0.061 |
|  | Interaction | 23.88 | 1.0 | 0.371 | 0.544 |
| Contractile Strain | Recurrence | 605.21 | 1.0 | 9.051 | 0.004 |
|  | Paroxysmal AF | 352.31 | 1.0 | 5.269 | 0.025 |
|  | Interaction | 10.69 | 1.0 | 0.160 | 0.691 |
| Reservoir Strain Rate | Recurrence | 129.67 | 1.0 | 3.872 | 0.053 |
|  | Paroxysmal AF | 255.47 | 1.0 | 7.629 | 0.007 |
|  | Interaction | 1.18 | 1.0 | 0.035 | 0.851 |
| Conduit Strain Rate | Recurrence | 62.76 | 1.0 | 1.507 | 0.224 |
|  | Paroxysmal AF | 276.41 | 1.0 | 6.636 | 0.012 |
|  | Interaction | 46.84 | 1.0 | 1.125 | 0.293 |
| Contractile Strain Rate | Recurrence | 516.44 | 1.0 | 11.601 | 0.001 |
|  | Paroxysmal AF | 287.38 | 1.0 | 6.455 | 0.013 |
|  | Interaction | 4.62 | 1.0 | 0.104 | 0.748 |

### Table 19: 2x2 ANOVA found no significant interaction effect between AF recurrence and AF type which suggests, despite the existence of group overlap, this had no substantial effect on the detected differences between AF type and AF recurrence separately.

**7.2 Logistic Regression Analyses**

| **Independent variables** | **coef** | **std err** | **z** | **P-value** | **Exp(coef)** | **Lower 95% CI** | **Upper 95% CI** |
| --- | --- | --- | --- | --- | --- | --- | --- |
| Reservoir strain | -0.068 | 0.022 | -3.133 | 0.002 | 0.934 | 0.896 | 0.975 |
| Paroxysmal AF | 0.755 | 0.614 | 1.230 | 0.219 | 2.128 | 0.639 | 7.086 |
| Conduit strain | -0.076 | 0.037 | -2.065 | 0.039 | 0.927 | 0.862 | 0.996 |
| Paroxysmal AF | -0.123 | 0.506 | -0.242 | 0.808 | 0.884 | 0.328 | 2.385 |
| Contractile strain | -0.118 | 0.036 | -3.289 | 0.001 | 0.889 | 0.828 | 0.953 |
| Paroxysmal AF | 0.640 | 0.564 | 1.135 | 0.257 | 1.897 | 0.628 | 5.732 |
| Reservoir strain rate | -0.129 | 0.047 | -2.776 | 0.006 | 0.879 | 0.802 | 0.963 |
| Paroxysmal AF | 0.509 | 0.602 | 0.845 | 0.398 | 1.663 | 0.511 | 5.414 |
| Conduit strain rate | 0.1110 | 0.050 | 2.241 | 0.025 | 1.117 | 1.017 | 1.231 |
| Paroxysmal AF | 0.135 | 0.569 | 0.237 | 0.813 | 1.144 | 0.375 | 3.494 |
| Contractile strain rate | 0.160 | 0.047 | 3.403 | 0.001 | 1.174 | 1.070 | 1.288 |
| Paroxysmal AF | 0.830 | 0.588 | 1.410 | 0.159 | 2.292 | 0.723 | 7.262 |

### Table 20: Logistic regression analyses using AF recurrence as the dependent variable. Independent variables: strain metric and paroxysmal AF. Paroxysmal AF was not a predictor of recurrence.

| **Independent variables** | **coef** | **std err** | **z** | **P-value** | **Exp(coef)** | **Lower 95% CI** | **Upper 95% CI** |
| --- | --- | --- | --- | --- | --- | --- | --- |
| Reservoir strain | 0.070 | 0.030 | 2.340 | 0.019 | 0.933 | 0.880 | 0.989 |
| Paroxysmal AF | 0.706 | 0.826 | 0.854 | 0.393 | 2.025 | 0.401 | 10.219 |
| Interaction | 0.004 | 0.044 | 0.090 | 0.929 | 1.004 | 0.922 | 1.093 |
| Conduit strain | 0.107 | 0.056 | 1.902 | 0.057 | 0.898 | 0.805 | 1.003 |
| Paroxysmal AF | 0.425 | 0.617 | 0.690 | 0.490 | 0.654 | 0.195 | 2.188 |
| Interaction | 0.059 | 0.074 | 0.805 | 0.421 | 1.061 | 0.919 | 1.226 |
| Contractile strain | 0.155 | 0.069 | 2.258 | 0.024 | 0.857 | 0.749 | 0.980 |
| Paroxysmal AF | 0.379 | 0.666 | 0.568 | 0.570 | 1.460 | 0.396 | 5.392 |
| Interaction | 0.058 | 0.082 | 0.704 | 0.482 | 1.059 | 0.902 | 1.244 |
| Reservoir strain rate | 0.167 | 0.073 | 2.273 | 0.023 | 0.847 | 0.733 | 0.977 |
| Paroxysmal AF | 0.130 | 0.780 | 0.167 | 0.867 | 1.139 | 0.247 | 5.258 |
| Interaction | 0.071 | 0.097 | 0.742 | 0.458 | 1.075 | 0.889 | 1.299 |
| Conduit strain rate | 0.111 | 0.066 | 1.685 | 0.092 | 1.118 | 0.982 | 1.272 |
| Paroxysmal AF | 0.131 | 0.773 | 0.170 | 0.865 | 1.140 | 0.251 | 5.189 |
| Interaction | 0.001 | 0.100 | 0.007 | 0.994 | 0.999 | 0.822 | 1.215 |
| Contractile strain rate | 0.207 | 0.093 | 2.224 | 0.026 | 1.230 | 1.025 | 1.476 |
| Paroxysmal AF | 0.577 | 0.689 | 0.836 | 0.403 | 1.780 | 0.461 | 6.873 |
| Interaction | 0.072 | 0.110 | 0.654 | 0.513 | 0.931 | 0.751 | 1.154 |

**Table 21:** Logistic regression analyses using AF recurrence as the dependent variable. Independent variables: strain metric, paroxysmal AF and multiplicative interaction (strain metric X paroxysmal AF). Neither paroxysmal AF nor the multiplicative interaction were predictors of recurrence.

**Supplementary References**

40. Shi, Wenzhe, et al. "Temporal sparse free-form deformations." *Medical image analysis* 17.7 (2013): 779-789.
